# Computational mining of MHC class II epitopes for the development of universal immunogenic proteins

**DOI:** 10.1371/journal.pone.0265644

**Published:** 2022-03-29

**Authors:** Kyle Saylor, Ben Donnan, Chenming Zhang

**Affiliations:** Department of Biological Systems Engineering, Virginia Tech, Blacksburg, Virginia, United States of America; University of Tennessee, UNITED STATES

## Abstract

The human leukocyte antigen (HLA) gene complex, one of the most diverse gene complexes found in the human genome, largely dictates how our immune systems recognize pathogens. Specifically, HLA genetic variability has been linked to vaccine effectiveness in humans and it has likely played some role in the shortcomings of the numerous human vaccines that have failed clinical trials. This variability is largely impossible to evaluate in animal models, however, as their immune systems generally 1) lack the diversity of the HLA complex and/or 2) express major histocompatibility complex (MHC) receptors that differ in specificity when compared to human MHC. In order to effectively engage the majority of human MHC receptors during vaccine design, here, we describe the use of HLA population frequency data from the USA and MHC epitope prediction software to facilitate the *in silico* mining of universal helper T cell epitopes and the subsequent design of a universal human immunogen using these predictions. This research highlights a novel approach to using *in silico* prediction software and data processing to direct vaccine development efforts.

## Introduction

The human leucocyte antigen (HLA) system, located on the short arm of chromosome 6, contains many genes that are essential to the initiation and maintenance of our immune systems. Specifically, the genes that code for major histocompatibility complex (MHC) class I molecules (HLA-A, HLA-B, and HLA-C) and MHC class II molecules (HLA-DP, HLA-DM, HLA-DO, HLA-DQ, and HLA-DR) reside here. These molecules activate cytotoxic T cells (T_C_ cells, via MHC I pathway) or helper T cells (T_H_ cells, via MHC II pathway) when abnormal intracellular or extracellular polypeptide species, respectively, are encountered and presented on the surface of any nucleated cell (in the case of MHC class I pathway) or specifically on antigen presenting cells (APCs, in the case of MHC class II pathway). In fact, the majority of adaptive immune system effector functions must be initiated by an MHC pathway at some point in time [1].

It is easy to understand how the evolutionary pressures placed on these pathways have culminated in one of the most diverse gene systems in the human genome. While this grand level of diversity is extremely beneficial when considering species survival, it becomes less beneficial on an individual basis when designing and implementing immunotherapies [2]. HLA allele variation has been implicated in malignancies, infections, and immunotherapeutic outcomes in many independent studies [3]. Some examples include the association of a specific allele with the occurrence of cancer, the association of HLA-DRB1 heterozygosity with better outcomes in viral infections, and the association of HLA class I homozygosity with checkpoint inhibitor inefficacy in cancer therapies [4–7]. Particularly in the case of vaccines, significant associations between HLA haplotype and vaccine outcome have been observed [8–11]. Causation in these studies, however, has not been established and it is important to note that other genes have also been linked to vaccine inefficacy [12]. Nonetheless, a new age of vaccine design has been born around the targeting of MHC molecules with epitopes that have been derived either experimentally or computationally using models based on pooled *in vivo* and *in vitro* data [13,14].

In any vaccine, it is possible that an antigen (or antigens) will lack epitopes specific for host MHC receptors. Without the loading of processed epitope onto MHC and the subsequent presentation of the peptide-MHC complex to T cell receptors (TCRs), there can be no activation of T_H_ and/or T_C_ cells. Consequently, the adaptive immune response to a vaccine in such situations will be severely impaired [15]. However, as the knowledgebase concerning these MHC activation systems continues to expand and mature, it is becoming more and more feasible and prudent to use all of the information we have to insure (in the case of vaccines) or prevent (in the case of biologics) the activation of T cells and the resultant adaptive immune response.

Many converging factors support the implementation of such an approach in the field of vaccinology. First, considerable variability in subjects’ response to a particular vaccine is a common occurrence and has been a deciding factor in the ultimate failure of some clinical trials [16–23]. The inevitability of acquiring variable outcomes when dealing with variable systems, however, can possibly be avoided by applying a more personalized approach to vaccination, particularly one that targets a large number of MHC molecules. Second, interspecies differences in HLA genotype and MHC specificity present as serious confounding elements when attempting to use animal studies as corollaries to success in future clinical trials [24]. Consequently, considering the MHC epitope content of antigen(s) prior to human studies may provide more insight into the likely outcome of clinical trials. Third, ample amounts of easily accessible software and data now exist online that can support an *in silico* approach to vaccine design. In fact, others have already begun the modulation of antigen immunogenicity based on these resources [25–27]. Last, there is an ever-present need to minimize the use of *in vivo* studies and adopt alternative vaccine quantitative methods. While animal studies are critical in evaluating the efficacy of a vaccine, *in silico* design and/or evaluation of vaccine candidates prior to human studies provides a plausible and acceptable alternative to *in vivo* testing.

In this study, common immunogen primary sequences were mined for HLA-DR and HLA-DQ epitopes using HLA population frequency data and MHC class II epitope prediction software. Other important human isotypes, such as the HLA-DP isotype, that code for MHC class II molecules were not included in this study due to lack of available epitope prediction methods and/or lack of sufficient population frequency data [13,28]. Murine IAd and IEd isotypes were analyzed in order to make inter-method and inter-species prediction output comparisons. MHC class II epitopes were chosen for analysis due to their role as facilitators of humoral immune responses via the activation of T_H_ cells. This activation is crucial to antibody production and as such plays an important role in the success of vaccines that are dependent on antibody effector functions, such as vaccines against drugs of abuse [29,30]. Using the output from these predictions, ‘universal’ chimeric antigens (UCAs) were designed that can be used to target mouse models with IAd and/or IEd isotype, and using HLA-DQB1 and HLA-DRB1 allele frequency data, 99% of the US population based on HLA-DQ and HLA-DR isotypes. To the best of our knowledge, this represents the first to attempt to design UCAs using HLA population frequencies and MHC class II epitope predictions. By making slight modifications to the approach, these UCAs could also be used to target certain demographics and/or individual subjects.

## Materials and methods

### HLA-DQB1 and HLA-DRB1 frequency analysis

Human HLA-DQB1 and HLA-DRB1 allele frequency data with race/ethnic associations were acquired from the National Bone Marrow Transplant (NBMW) / Be The Match bioinformatics database [28]. Data were sorted based on overall population frequency, and the most common beta alleles accounting for at least 99% of the database test population were selected as targets for MHC class II epitope predictions. Since only population frequency data for beta chains were available, alpha-beta pairing was done post-hoc and did not represent population frequencies for complete MHC class II molecules (which consist of one alpha chain and one beta chain and can be subject to various levels of genetic linkage).

### Source immunogen selection

The most common immunogens used in vaccine formulations were identified from a thorough literature review. Some of these have been approved for use in human vaccines by the FDA, such as diphtheria toxoid (DT), tetanus toxoid (TT), and the HPV 16 L1 protein (HPV). Others, such as keyhole limpet hemocyanin 1 and keyhole limpet hemocyanin 2 (KLH1 and KLH2) and bovine serum albumin (BSA) are typically only used in proof-of-concept, animal studies. Human serum albumin (HSA) and mouse serum albumin (MSA) were selected as benchmark proteins for the analysis of the other selected immunogens. In total, the 15 most common immunogens encountered during the literature review were chosen as MHC class II epitope sources. In addition to the six immunogens already mentioned, the additional nine were the Q-beta capsid protein (QB), *Pseudomonas aeruginosa* exoprotein A (EPA), cholera toxin subunit B (CTB), heat labile enterotoxin B (LTB), outer membrane protein C (OMPC), Influenza A hemagglutinin (HA), hepatitis B core antigen (HBC), MS2 capsid protein (MS2), and hepatitis C core antigen (HCC). Sequences were obtained from the UniProt website [31]. A summary of these immunogens can be found in Table 1.

**Table 1:** Common immunogen information.

Protein	Abbr.	Residues	UniProt Source
Cholera toxin subunit B	CTB	104	Q55DA8
Heat labile enterotoxin B	LTB	124	A05XG5
MS2 capsid protein	MS2	130	P03612
Q-beta capsin protein	Qb	133	P03615
Hepatitis C core antigen	HBcAg	150	Q68842
Hepatitis B core antigen	HBcAg	185	P03148
Influenza A hemogglutinin	HA	328	P04664
Outer membrane protein C	OMPC	367	C6K7N1
Human papillomavirus 16 L1	HPV16L1	505	A0A161GUX4
Diphtheria toxin	DT	560	Q6NK15
Bovine serum albumin	BSA	607	P02769
Murine serum albumin	MSA	608	P07724
Human serum albumin	HAS	609	P02768
Pseudomonas aeruginosa exoprotein A	EPA	638	P11439
Tetanus toxin	TT	1315	P04958
Keyhole limpet hemocyanin 1	KLH1	3125	Q6KC56
Keyhole limpet hemocyanin 2	KLH2	3421	Q6KC55

### MHC class II epitope predictions

MHC class II epitope predictions were performed using software downloaded from the Immune Epitope Database and Analysis Resource (IEDB.org, v2.22.1) [13]. Specifically, predictions were run for each immunogen-isotype pairing using neural network-based NetMHCIIPan 3.2 method (DQ, DR, and IAd predictions) and the stabilization matrix alignment-based SMM-align method (IAd and IEd predictions) [32,33]. Both of these prediction methods have been shown to have high accuracy in the past [34,35]. IAd and IEd predictions were included in the analysis in order to 1) compare between prediction methods (NetMHCIIPan vs. SMM-align), 2) compare between species (human and mouse), and 3) set up for *in vivo* evaluation of this immunogen design approach. An additional parameter, peptide length, was specified as 15–20 amino acids for the predictions. This range of epitope lengths (which falls within the 9–23 residue range of lengths defined for most MHC class II epitopes) was chosen in order to introduce diversity into the prediction, thus helping to eliminate any output bias that might be due to input of a single epitope length [36].

### Epitope scoring and anchor residue identification

Epitope scoring was achieved by completing an unweighted summation (no core residue bias) of transformed percentile rankings (100-x) for each amino acid within each predicted epitope. The final sums were normalized by dividing by the average residue score for the endogenous, HSA protein, and an ‘immunogenicity threshold’ was established at one for these normalized values. Alternately, MSA was used as the benchmark when establishing immunogenicity thresholds for the IAd and IEd predictions. Anchor residues, which are defined as the amino acids contained within an epitope that have a high affinity for cognate MHC class II molecule relative to their neighbors, were identified in a similar fashion [35]. The only differences between the two scoring mechanisms were that a weighted summation (four to one core residue to non-core residue weighting) and an ‘anchor residue’ threshold value of four were used for anchor residue identification purposes. Both the ‘unweighted, normalized, and cumulative’ (UNC) and ‘weighted, normalized, and cumulative’ (WNC) methods resulted in proteins being assessed based on the 13 HLA-DQB1 and 40 HLA-DRB1 alleles specified in the frequency analysis, even though predictions for the HLA-DQ epitopes also required the specification of an alpha chain. As a result, predictions made for HLA-DQ epitopes may not have targeted the most frequent alpha-beta pairs, though 99% of the sample population should have been targeted nonetheless. The mean and standard deviation for summed residue immunogenicity values were calculated for each protein using UNC and WNC results, respectively. These values were then plotted against residue number. All prediction output analyses were performed and all plots were generated using Matlab (ver. R2019a).

### Unweighted epitope score analyses, ranking, and excision

UNC outputs were subjected to a moving mean analysis (MMA, n = 13) of modified prediction values that incorporated both the mean (positive, indicated likely epitope region) and the standard deviation (negative, indicated intra-isotype discrepancies between prediction outputs) of the residue scores. The rational for the prediction value modification, which was achieved by subtracting 3x the standard deviation from the mean for each residue, was that regions within the analyzed immunogens most likely to display both immunogenicity AND promiscuity as MHC II epitopes were of most interest. Local maximums were identified within UNC MMAs and excised with 12 flanking residues on each side. These 25 amino acids long ‘epitope candidates’ were then ranked based on the summation of the scores of their composing residues. Additional data was also recorded, such as the epicenter residue location, the amino acid sequence, the local maximum value, and the number(s) and location(s) of the anchor residues present within the excised epitope candidate. Epitope scoring and excision was performed using Matlab (ver. R2019a) and ranking was performed using Microsoft Excel (ver. 1808).

### Conception and analysis of isotype-specific, universal immunogens

Chimeric proteins designed to maximize immunogenicity for HLA-DR, HLA-DQ, IAd, and IEd isotypes were constructed by concatenating the twenty highest scoring epitopes from post-prediction UNC MMAs with interspacing di-glycine-lysine linkers (for hapten attachment, KGGKGGK) flanked by cathepsin S-sensitive sequences (to facilitate processing by APCs, GGVVRGG) [37,38]. It is important to note that the inclusion of cathepsin S-sensitive sequences should promote site-specific proteolysis, though the extent of antigenic processing is largely beyond the control of initial immunogen design due to the presence of multiple protease species (all characterized by varying levels of specificity and activity) in lysosomal compartments [39,40]. Host-derived epitopes and those that lacked predicted anchor residues, however, were removed from the ranking pool. Using a similar approach, non-antigenic, chimeric proteins (UCnAs) were engineered by replacing the highest scoring epitopes with those that had the lowest UNC MMA scores. For comparative purposes, a random protein sequence that was the same length as the antigenic and non-antigenic chimeric proteins was also generated in Matlab and analyzed in parallel with the UCAs and UCnAs. All three of these proteins (the antigenic, non-antigenic, and random) were then processed using the epitope prediction, scoring, and analysis methods previously described. Results from this step were used to generate line charts based on means (and standard deviations, if available) for all UCAs, UCnAs, and the random protein and heat maps for final HLA-DR and HLA-DQ isotype-specific outputs in Matlab (ver. R2019a).

### Comparing prediction methods and outputs

**Fig 1:**
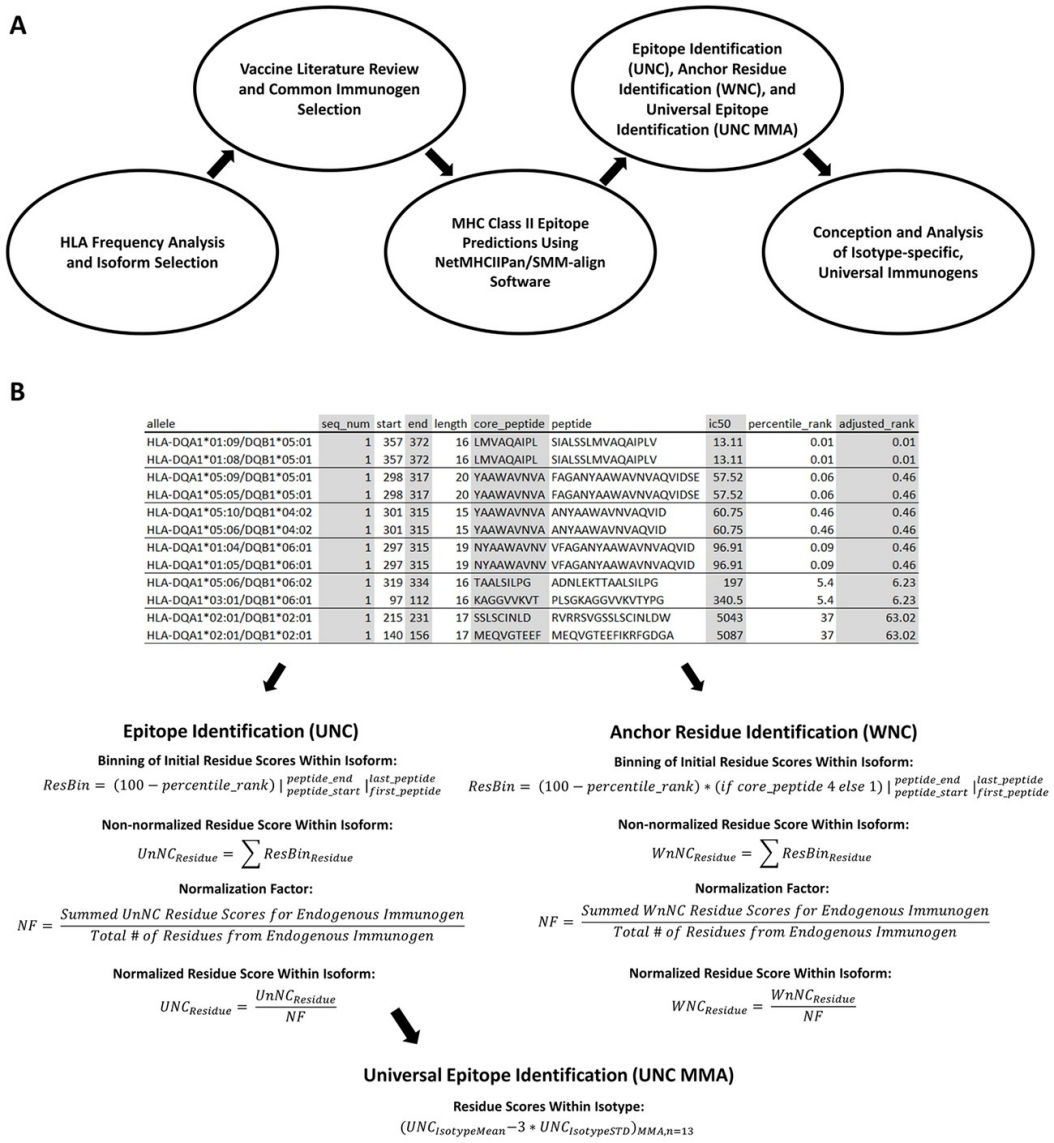
Schematic overview of methodology. (A) The overall study methodology and (B) the epitope scoring methodology. In the epitope scoring methodology, excerpts of NetMHCIIPan prediction output for the HLA-DQ/DT isotype/immunogen pairing are provided for reference. Binning of residue scores (calculated using epitope predictions) was achieved via iteration through epitope residues and epitopes within an isoform. This was the first step in both the UNC and WNC analysis and was performed for each isoform. Non-normalized residue scores (UnNC and WnNC) were then calculated by summing the score components of each residue bin. Normalized scores were calculated by dividing the non-normalized values by the average non-normalized residue scores for an endogenous immunogen (either HSA or MSA, depending upon the prediction isotype). UNC MMA scores were calculated by taking the mean of normalized residue scores from all isoforms within an isotype, subtracting three standard devi-ations, and applying a moving average (n = 13).

Differences between method/isotype-specific prediction outputs were quantified using three methods. The first approach involved taking the difference between mean residue scores (averaged over all of the isoforms within the isotype) and then subsequently finding the overall mean and standard deviation of these values. This method was performed for all method/isotype combinations. The second method involved taking the absolute value of the difference between mean residue scores (averaged over all of the isoforms within the isotype) and then subsequently finding the overall mean and standard deviation of these values. This method was also performed for all method/isotype combinations. The third method involved finding the overall mean and standard deviation for all normalized, isotype-specific residues scores. Statistical comparisons were made, when possible, in SAS JMP Pro 14 using Tukey’s HSD method. A schematic overview of the methodologies followed here can be found in Fig 1A and 1B.

## Results

### Input data collection and setup

**Fig 2:**
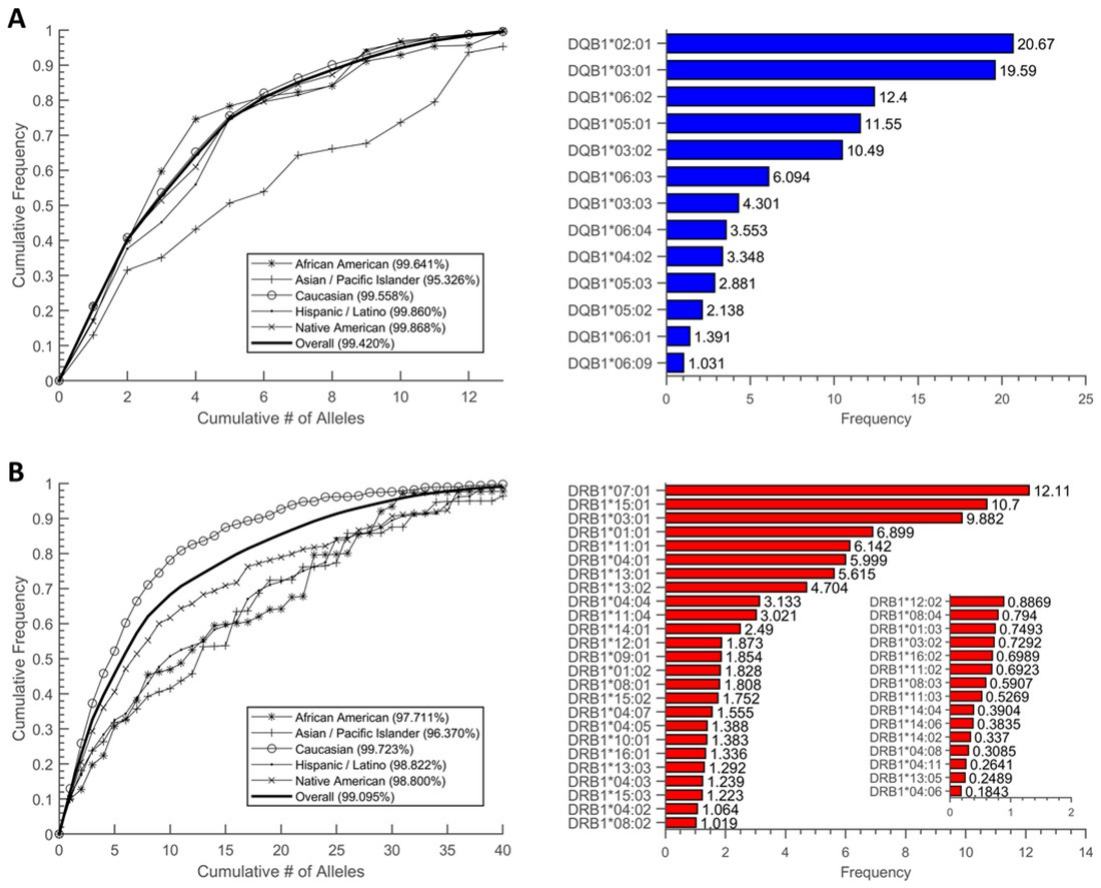
HLA population frequencies. (A) HLA-DQB1 and (B) HLA-DRB1 cumulative (line graphs with race/ethnicity data) and individual allele (bar graphs) population frequency information is displayed here. Cumulative frequency plots display summed frequencies of sequentially ordered HLA beta alleles (greatest to smallest for total population) vs. the number of alleles included in the sum. Individual allele plots display HLA alleles vs. their respective overall population frequency.

Common immunogens were successfully identified in the literature and their sequences were obtained from the UniProt website. Additionally, population frequency data for HLA-DQB1 and HLA-DRB1 alleles were sorted and target alleles were successfully identified. These frequencies, both cumulative and for each race/ethnicity, are shown in Fig 2A and 2B for HLA-DQB1 and HLA-DRB1 alleles, respectively. A tabulated version of this data can also be found in S1 Table. In total, the top 13 HLA-DQB1 alleles were necessary in order to achieve 99% total population coverage. With 28 possible HLA-DQA1 chains, the total number of MHC class II isoforms that were needed for the HLA-DQ prediction input was therefore 364. On the other hand, the top 40 HLA-DRB1 alleles were necessary in order to achieve 99% total population coverage. With only one possible HLA-DRA1 chain, the total number of MHC class II isoforms that were needed for the HLA-DR prediction input remained at 40. Since IAd and IEd are single isoforms that represent complete HLA isotypes, their epitope prediction runs only required a single input.

### MHC class II epitope predictions, scoring, and analysis

In total, 6,919 epitope predictions were necessary in order to achieve target population coverage. The breakdown for this total was 6,188 HLA-DQ predictions (364x17), 680 HLA-DR predictions (40x17), and 17 IAd (NetMHCIIPan), IAd (SMM), and IEd predictions each. All of these epitope predictions were completed successfully, though there were sizeable variations in prediction run times due to differences in protein length and isoform input requirements.

**Fig 3:**
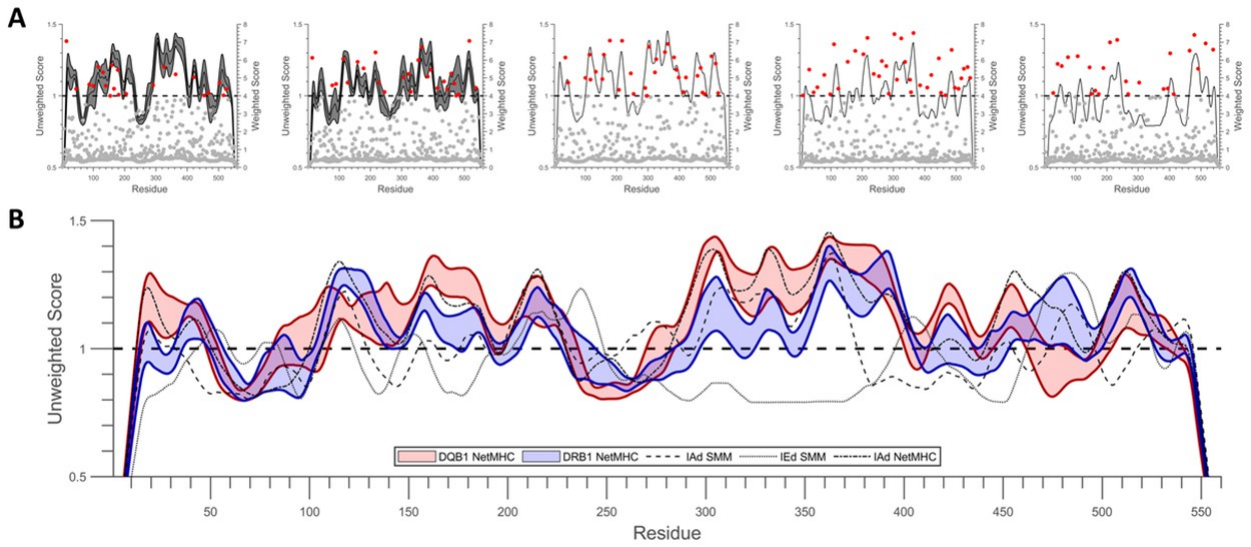
HLA-DQ/DT epitope analysis results. (A) DT epitope scoring (UNC) and anchor residue identification (WNC) results for the HLA-DQ isotype. Scores, in line form (black, UNC) or dot form (red, WNC), are plotted against residue number. For HLA-DQ (1st from left) and HLA-DR (2nd from left) results, the center line represents the mean score and the shaded area represents ±1 standard deviation. For IAd NetMHC (3rd from left), IAd SMM (4th from left), and IEd SMM (5th from left) results, lines represent the mean score. (B) DT UNC results for all isotypes are plotted together here for easier comparison. Scores are plotted in line form against residue number. For HLA-DQ and HLA-DR results, the shaded area represents the mean score ±1 standard deviation. For IAd NetMHC, IAd SMM, and IEd SMM results, lines represent the mean score. In both parts, shaded areas representing standard deviation could not be incorporated with the IAd and IEd results due to lack of diversity within the isotypes (these plots summarize a single immunogen/isoform prediction run). UNC, WNC, and combined results for other isotype/immunogen combinations can be found in Supporting Information.

Epitope scoring (using UNC prediction outcomes) and anchor residue identification (using WNC prediction outcomes) were also completed successfully. Line plots (and dot plots) of the unweighted (and weighted) results from this step for each immunogen/isotype pairing can be found in S1 and S2 Figs. Plots that combined the UNC results of all isotypes within a single plot for each immunogen can be found in S3 Fig. An example of the prediction and analysis outputs, using DT as the model immunogen, can be found in Fig 3. According to both raw and processed prediction output, all of the immunogens analyzed contained at least one MHC class II epitope. Additionally, the WNC analysis results revealed that each immunogen contained multiple predicted anchor residues. Inter-species, inter-isotype, and inter-method differences were all observed when comparing UNC and WNC outputs for each immunogen. A summary of the prediction, scoring, and analysis results can be found in Table 2. Within the table, ‘hits’ indicate the number of outputs generated by the prediction software and ‘epitopes’ indicate the number of protein regions determined to be immunogenic post-UNC processing.

**Table 2:** Overview of prediction outcomes.

Protein	Residues	DR Hits	DR Epitopes	DQ Hits	DQ Epitopes	IAd(Net) Hits	IAd(Net) Epitopes	IAd(SMM) Hits	IAd(SMM) Epitopes	IEd(SMM) Hits	IEd(SMM) Epitopes
Cholera toxin subunit B	104	21000	2	191100	3	525	2	525	3	525	2
Heat labile enterotoxin B	124	25800	3	234780	3	645	3	645	3	645	2
MS2 capsid protein	130	27240	3	247884	2	681	2	681	2	681	3
Q-beta capsin protein	133	27960	3	254436	3	699	2	699	2	699	2
Hepatitis C core antigen	150	32040	3	291564	4	801	3	801	3	801	2
Hepatitis B core antigen	185	40440	4	368004	3	801	3	1011	4	1011	5
Influenza A hemogglutinin	328	74760	8	680316	7	1869	7	1869	7	1869	8
Outer membrane protein C	367	84120	9	765492	8	2103	8	2103	9	2103	9
Human papillomavirus 16 L1	505	117240	12	1066884	11	2931	13	2931	12	2931	9
Diphtheria toxin	560	130440	15	1187004	16	3261	13	3261	15	3261	12
Bovine serum albumin	607	141720	13	1289652	16	3543	14	3543	11	3543	14
Murine serum albumin	608	141960	13	1291836	18	3549	14	3549	13	3549	13
Human serum albumin	609	142200	13	1294020	15	3555	14	3555	11	3555	15
Pseudomonas aeruginosa exoprotein A	638	149160	18	1357356	17	3729	17	3729	16	3729	15
Tetanus toxin	1315	311640	31	2835924	33	7791	29	7791	28	7791	27
Keyhole limpet hemocyanin 1	3125	746040	77	6788964	78	18651	73	18651	67	18651	67
Keyhole limpet hemocyanin 2	3421	817080	82	7435428	96	20427	85	20427	73	20427	79

As expected, MMA of the UNC results revealed discrepancies between HLA-DQ and HLA-DR isotype-specific epitope predictions. There were also stretches within the target immunogens’ primary sequence that were shown to have a considerable degree of intra-isotype (and sometimes even inter-isotype) promiscuity. For example, multiple regions of diphtheria toxin were found to be highly promiscuous and as such were included in the isotype-specific UCA design and analysis process. Among these, the highest rated HLA-DQ 25-mer epitope was found centered at residue 366. An example of the final epitope identification and excision results (those for the HLA-DQ isotype) can be found in Table 3. All other results (i.e. those for the HLA-DR, IAd NetMHC, IAd SMM, and IEd SMM isotypes/methods) can be found in S2 Table.

**Table 3:** HLA-DQ epitope ranking and excision results.

Protein	Epicenter	Residues	Peak Score	Cummulative Score	Anchor(s)	Anchor Location(s)
DT	366	VAQSIALSSLMVAQAIPLVGELVDI	1.244903473	30.00208733	1	363
KLH2	3116	LWLGGTETYSMSSLAFSAYDPVFMI	1.266599245	29.66917927	2	3113;3128
KLH1	2126	LKYALSSLQADTSADGFAAIASFHG	1.203760532	28.79987579	3	2116;2127;2133
TT	683	VLLLEYIPEITLPVIAALSIAESST	1.18984899	28.47710714	1	685
KLH2	3230	LRNQPRVFAGFVLSGIYTSANVKIY	1.155509004	28.26449885	1	3228
EPA	470	GYVFVGYHGTFLEAAQSIVFGGVRA	1.195748733	28.07205997	1	464
KLH2	3030	IPYWDWTKSMIALPAFFADSSNSNP	1.220294602	27.98888619	1	3024
KLH1	1714	ESMKADHSSDGFQAIASFHALPPLC	1.18117356	27.94054821	2	1713;1716
HA	113	DVPDYASLRSLVASSGTLEFITEGF	1.178072602	27.89447046	3	105;112;125
KLH1	752	EDRIYAGFLLAGIRTSANVDIFIKT	1.194509058	27.84109025	2	747;752
KLH2	330	RAAKERTFASFILSGFGGSANVVVY	1.1450165	27.76877714	1	341
KLH1	1995	QEHSRVFAGFLLEGFGTSATVDFQV	1.194575351	27.7396872	2	1983;1994
EPA	496	SQDLDAIWRGFYIAGDPALAYGYAQ	1.157977165	27.72034364	2	491;502
KLH2	2823	KEERTFAAFLLHGFGASADVSFDVC	1.19423916	27.50198956	2	2813;2821
MSA	243	GERAFKAWAVARLSQTFPNADFAEI	1.191126486	27.48352048	1	232
EPA	182	LARDATFFVRAHESNEMQPTLAISH	1.119360222	27.44939067	2	175;192
KLH1	1038	YEIAHNYIHALVGGAQPYGMASLRY	1.140683141	27.35522704	3	1028;1035;1048
TT	268	KQEIYMQHTYPISAEELFTFGGQDA	1.133009845	27.30085116	2	264;272
OMPC	285	WANKAQNFEAVAQYQFDFGLRPSLA	1.148137149	27.2273307	1	289
DT	159	EFIKRFGDGASRVVLSLPFAEGSSS	1.130103304	27.11920376	3	154;159;166
DT	69	QKGIQKPKSGTQGNYDDDWKGFYST	0.750778926	18.86486125	0	-
KLH2	3285	FKYDITEVANRLNMHHDDTFNFRLE	0.710019791	18.6720013	0	-
MSA	275	TKVNKECCHGDLLECADDRAELAKY	0.675856369	18.60844917	0	-
OMPC	226	IGGAISSSKRTDAQNTAAYIGNGDR	0.702058163	18.58162638	0	-
MSA	127	CTKQEPERNECFLQHKDDNPSLPPF	0.72994394	18.41383759	0	-
KLH2	2050	QFDRLYKYDITKTLKDMKLRYDDTF	0.656917303	18.34949986	0	-
QB	74	NYKVQVKIQNPTACTANGSCDPSVT	0.676758453	18.24156848	0	-
TT	1172	GKLNIYYRRLYNGLKFIIKRYTPNN	0.663503833	18.18220649	0	-
BSA	127	CEKQEPERNECFLSHKDDSPDLPKL	0.709074042	17.90439636	0	-
BSA	329	EKDAIPENLPPLTADFAEDKDVCKN	0.682086932	17.8524391	0	-
TT	331	IDSYKQIYQQKYQFDKDSNGQYIVN	0.69292518	17.80870386	0	-
HA	77	TLIDALLGDPHCDVFQDETWDLFVE	0.651700228	17.75576302	0	-
TT	487	LTFIAEKNSFSEEPFQDEIVSYNTK	0.682363508	17.73314233	0	-
HPV	173	CKPPIGEHWGKGSPCTNVAVNPGDC	0.654750628	17.56775931	0	-
HCV	44	YLLPRRGPRLGVRATRKTSERSQPR	0.6604623	17.24324764	0	-
HSA	280	ECCHGDLLECADDRADLAKYICENQ	0.637717696	17.00537164	0	-
HSA	408	KVFDEFKPLVEEPQNLIKQNCELFE	0.632871202	16.65259624	0	-
KLH1	262	DCAQELLHQKMEPFSWEDNDIPLTN	0.631582056	16.4462453	0	-
BSA	280	CCHGDLLECADDRADLAKYICDNQD	0.619778655	16.39374163	0	-
KLH2	1217	WRYDRVYKYEITQQLHDLDLHVGDN	0.602212809	15.85052422	0	-

**Epicenter**—location (residue number) of epitope center in relation to parent protein sequence.

**Peak score**—highest residue UNC score found within the indicated epitope.

**Cummulative score—**the summation of UNC scores for all the residues found within the indicated epitope.

**Anchor**—a residue that achieved a WNC score of >4; indicates a residue with particular importance in MHC interactions.

**Anchor locations**—location (residue number) of the anchor residues in relation to the parent protein sequence.

### Isotype-specific UCA design and analysis results

**Fig 4:**
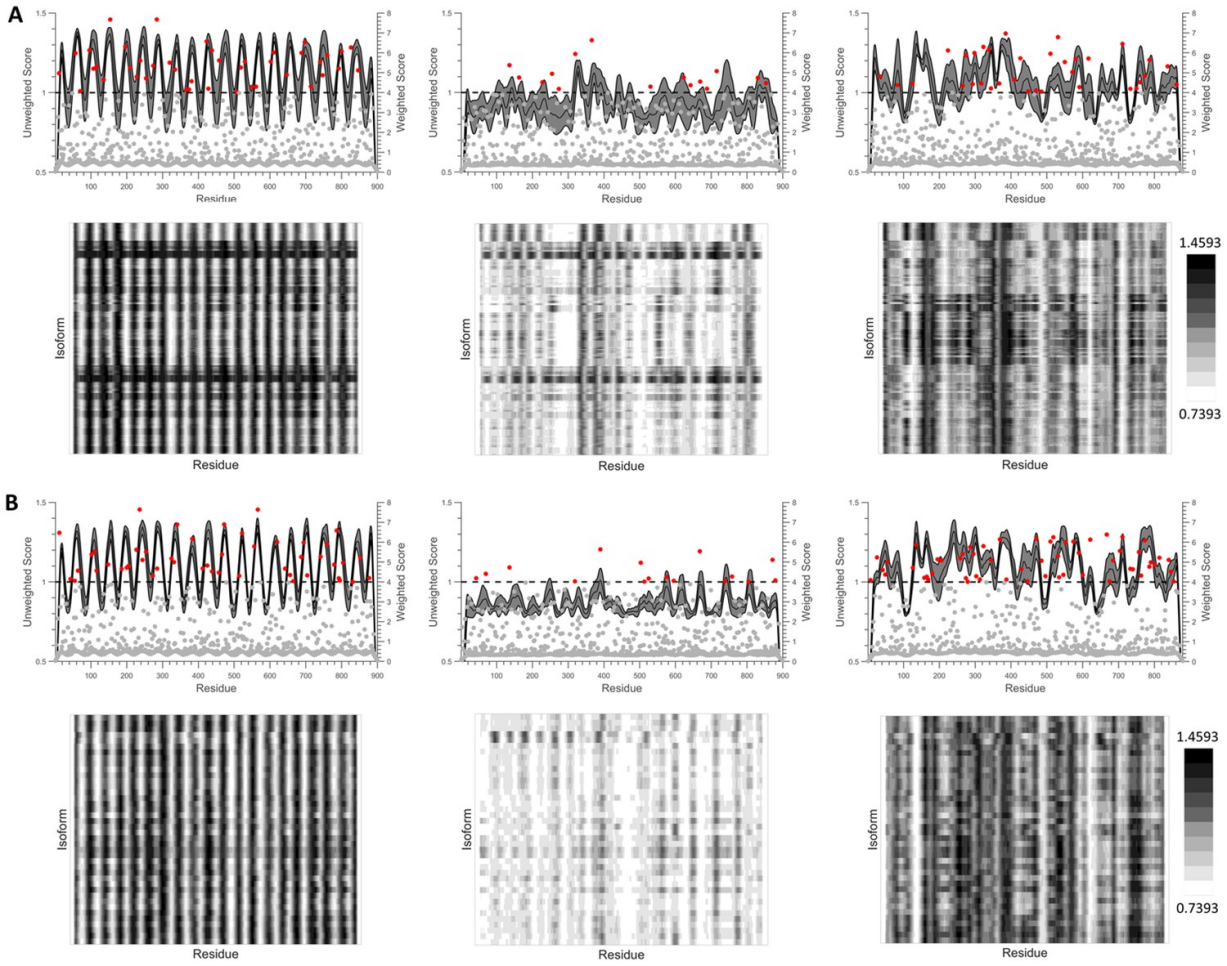
Design and assessment of DQ- and DR-specific UCAs and UCnAs. The plots and heat maps shown here summarize epitope scores for (A) HLA-DQ and (B) HLA-DR isotypes. Top plots display scores for UCAs (1st from left), UCnAs (2nd from left), and a random protein of the same length (3rd from left), in line form (black, UNC) or dot form (red, WNC), plotted against residue number. Center lines represent mean scores and shaded areas represent ±1 standard deviation. Bottom heat maps display isoform vs. residue number for UCAs (1st from left), UCnAs (2nd from left), and a random protein of the same length (3rd from left). Lighter regions and darker regions within the heat map represent lower and higher immunogenicity scores, respectively.

The top twenty epitopes identified for each HLA isotype were successfully used to generate an isotype-specific UCA. Isotype-specific universal chimeric non-antigens (UCnAs) were also successfully generated for each HLA isotype using the twenty least immunogenic 25-mer amino acid stretches found in the UNC MMA results. Analysis of the UNC and WNC results for the UCAs and UnCAs, in addition to the random sequence protein of equal size, indicated that promising epitopes (when considering predicted immunogenicity) had been successfully identified within each immunogen for all of the isotypes included in the study. Finally, analysis of the isotype-specific HLA-DQ and HLA-DR UNC results using heat maps revealed considerable intra-isotype overlap between epitope predictions. The HLA-DQ and HLA-DR results from these analyses can be found in Fig 4A and 4B, respectively. For the IAdNetMHC, IAdSMM, and IEdSMM results, please refer to S4 Fig.

### Inter-method comparisons

**Fig 5:**
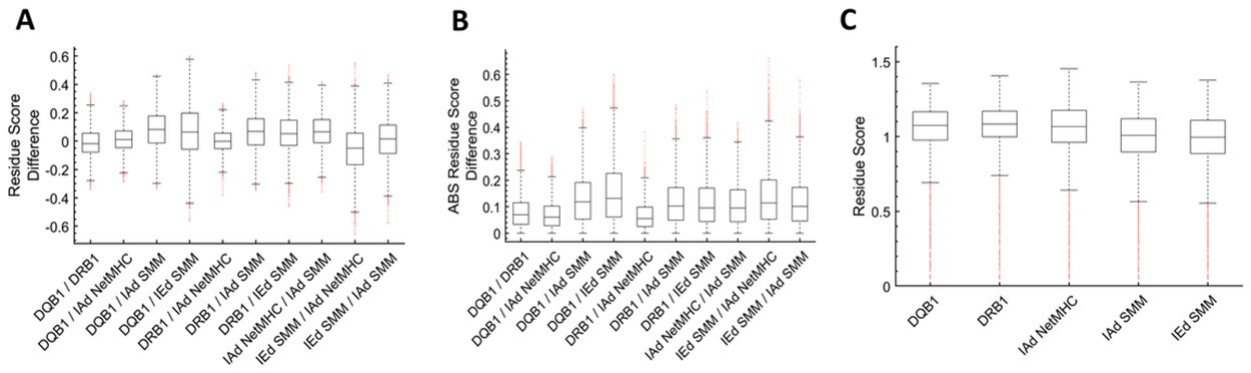
Comparing between prediction methods. (A) Direct comparison of differences in individual residue scores (when necessary, averaged over all isoforms within an isotype) between isotypes/methods in boxplot format (first indicated isotype/method minus second indicated isotype/method). (B) Comparison of the absolute value of the differences in individual residue scores (when necessary, averaged over all isoforms within an isotype) between isotypes/methods in boxplot format. (C) Comparison of residue scores of isotypes/methods in boxplot format.

When residue scores for all isoforms within and isotypes/methods were averaged, differences and comparisons between method/isotype-specific outputs were compared, and a difference could not be detected between the DQB1 and IAd NetMHC outputs (p = 0.05). These results can be seen in Fig 5A. General comparisons using the absolute value of the difference between mean residue scores, however, indicated that all of the method/isotype-specific outputs yielded different results on an individual residue basis. These results can be seen in Fig 5B. Interestingly, the comparison between NetMHCIIpan and SMM-align methods (both were used to predict epitopes for the IAd isotype) yielded sizably different results, indicating that, at least when using the methodologies outlined here, the prediction outputs provided different results on an individual residue basis. Additionally, general comparison between method/isotype residue scores indicated that NetMHC and SMM prediction methods produced different results in relation to endogenous benchmark, with NetMHC results falling above the benchmark and SMM results falling below. These results can be found in Fig 5C.

## Discussion

The goal of this project was to create a chimeric, human immunogen based on the concatenation of conjugation sites, cathepsin cleavage sites, and computationally mined MHC class II epitopes that were predicted to 1) be highly immunogenic, 2) be highly promiscuous, and 3) contain at least one anchor residue. If enough of these putative epitopes were included within the primary structure of an engineered immunogen, it is likely that the immunogen would elicit a sizeable adaptive immune response in the majority of those inoculated. To the best of our knowledge, this strategy for potentiating conjugate vaccine immunogenicity has not been previously explored.

Any MHC epitope-based vaccine designed for human use would be impossible to satisfactorily evaluate outside of clinical trials (even when considering the plethora of humanized mouse models available for immunological research) [41,42]. As such, mouse HLA isotypes IAd and IEd were targeted within the study as well. In this way, the approach outlined for the development of a universal human immunogen could be evaluated in mice first. The development of an MHC epitope-rich, mouse immunogen and its pre-clinical evaluation would effectively allow the assessment of epitope prediction approaches to targeted conjugate vaccine development. Crucially, however, the isogenic nature of common laboratory mouse strains (both wild-type and humanized) does not allow for the assessment of targeting genetically diverse HLA populations with universal epitopes. For this purpose, human studies, or numerous binding assays, would still be necessary.

It is important to note that the origin of the sample population used to generate the HLA frequency data was limited to the United States of America (USA). This lack of geographical diversity, in addition to the over-representation of Caucasians within the dataset, is certainly not ideal. Race/ethnicity was accounted for within the data, however, so inferences about vaccine efficacy outside of the USA can be made. The lowest HLA-DQ allele set coverage exceeded 95% (Asian / Pacific Islander) and the lowest HLA-DR allele set coverage exceeded 96% (Asian / Pacific Islander). These numbers suggest that the HLA-DQ and HLA-DR vaccine candidates proposed in this study would also be effective in non-USA populations.

Common immunogens were chosen due to their success and safety in prior vaccine formulations. The success suggested that ample numbers of MHC class II epitopes were present in their primary structures and that the epitope prediction step would likely also be a success. It was possible that these immunogens lacked sufficiently immunogenic and promiscuous epitopes to create UCAs. This issue could have been overcome with the simple addition of more immunogens to the mining process. An approach that used random proteins would also have worked, but it would have required more computational power and time in order to yield the same number of high-potential, predicted epitope candidates. There was some concern regarding whether the use of epitopes specific for existing memory T cell pools would culminate in potentiation or regulation of immune response [43]. In the event that issues such as regulation are encountered during *in vivo* vaccine assessment, however, it is important to note that novel epitopes mined from randomly generated proteins could always be employed in place of common immunogen-derived epitopes.

The output data files from many of the HLA-DQ and HLA-DR predictions were immense and as such they were not reviewed in full prior to processing. Review of the first 200 lines of output for all prediction assignments, however, indicated acceptable data integrity. It was therefore assumed that any unreviewed output data would have similar, acceptable integrity. There were many additional caveats associated with the epitope and anchor residue scoring systems. First, overlapping epitope predictions (even those that had the same core residues) were interpreted as a positive outcome and should be incorporated in the final epitope analysis process. This interpretation is contrary to some of the recommendations received from IEDB staff that supported collecting only the top prediction output established for each unique core. Second, it was hypothesized that core residues that appeared more often within the prediction output would play a more sizeable role in epitope-MHC interaction. As such, codes were generated that could elucidate the presence of potential anchor residues (refer to the WNC analysis). While MHC anchor residues are an established phenomenon, there were no previous studies that could validate their identification [44]. Third, the chimeric antigen validation system presented here used nearly identical methodologies to that of the epitope ranking and anchor residue identification systems. As such, further epitope validation (both experimental and computational) should be performed in the future in order to better evaluate the immunogenicity and promiscuity assessments established using these methods.

Many interesting observations were made when analyzing the prediction outputs. UNC results indicated that most immunogens already contained regions with high immunogenicity scores. Further analysis of the HLA-DQ and HLA-DR UNC results, however, revealed that the majority of these regions had highly variable immunogenicity scores across different isotypes. This result indicated that, while all of the immunogens would most likely elicit a sizeable immune response in some people, responses would be variable, and variable vaccine responses are generally poor corollaries for success. In fact, as a side note, it would be interesting to see if these discrepancies correlated with past conjugate vaccine clinical failures, such as the Phase III trial for NicVAX that used the EPA immunogen, the Phase II trial for AngQb that used the QB immunogen, or the Phase II trial for TA-NIC that used the CTB immunogen [23,45].

When compared, the WNC analysis did not appear to completely coincide with the UNC analysis. That is, some anchor residues seemed to appear in a random manner throughout the length of each protein. This indicated that, while important, the anchor residues were not the deciding factor for epitope identification. Interestingly, some of the most immunogenic epitopes predicted for the IAd and IEd isotypes were from MSA. This result could indicate that epitope predictions include motifs that are cognate for regulatory T cells (T_Reg_ cells). If this is in fact the case, more work will need to be done in the future in order to accommodate our prediction assessments with a means of discriminating between epitopes cognate for T_H_ cells and T_Reg_ cells.

Analysis of the UNC and WNC results for the UCAs and UnCAs, in addition to the random sequence protein of equal size, indicated that promising epitopes (when considering predicted immunogenicity and promiscuity) had been successfully identified within each immunogen for all of the isotypes included in this study. It is important to note, however, that the serial concatenation of ranked polypeptides used in this study to generate UCAs and UnCAs may need re-evaluation in the future. Immune responses can be influenced by all levels of immunogen structure (primary, secondary, tertiary, and quaternary). It is possible that the linear organization of the epitopes and linkers proposed in this study constitutes or promotes a structural configuration that is unsafe or attenuates processing by antigen presenting cells (15). If animal studies or early clinical studies indicate inefficacy and/or high toxicity, rearrangement of the epitopes in the UCA would definitely be warranted.

The comparison of top scoring epitopes and general comparisons between prediction methods were able to identify similarities and discrepancies between various sub-groups. Comparison of NetMHCIIPan and SMM-align methods when considering IAd isotype predictions revealed differences on an individual residue basis and discrepancies between top-ranked epitopes. As such, any strategy for *in silico* vaccine design is recommended to incorporate predicted epitopes from various methods, if available. The inter-species differences observed when comparing both individual residue scores and top-ranking epitopes illuminate the inadequacy of using animal models to evaluate epitope-based vaccines designed specifically for humans. Alternatively, the inter-isotype similarities encountered in the UNC MMA may presage difficulties in establishing specific isotype/epitope effects on immunogenicity if human studies are ever conducted.

This paper describes the novel application of HLA population genetics and cumulative assessment of predictions to the variable immunogenicity, which plagued many conjugate vaccines in the past. In the future, this approach to epitope identification could also be used for more than just the development of a universal, chimeric immunogen. For example, the development of demographic-specific carrier proteins, individually personalized carrier proteins, and pathogen-specific, universal immunogens are all possible by using this method. In the later application, careful consideration of structural conformation would be warranted. Carrier proteins theoretically benefit from heterogeneous, higher-order structure (barring the event of inefficient hapten conjugation), as structural diversity discourages the formation of antigenic determinants that would compete with hapten. Any recombinant immunogen requiring self-provision of antigenic determinants, however, would likely benefit from efforts to insure homogeneous, higher-order structure that consistently displays the most relevant conformational epitopes.

## Supporting information

S1 Fig MHC epitope analysis results for immunogens/benchmarks 1–10. Epitope scoring (UNC) and anchor residue identification (WNC) results for individual isotypes / prediction methods are presented side-by-side. Scores, in line form (UNC) or dot form (WNC), are plotted against residue number. For HLA-DQ and HLA-DR results, the center line represents the mean score and the shaded area represents ±1 standard deviation. For IAd NetMHC, IAd SMM, and IEd SMM results, lines represent the mean score.
(PDF)

S2 Fig MHC epitope analysis results for immunogens/benchmarks 11–17. Epitope scoring (UNC) and anchor residue identification (WNC) results for individual isotypes / prediction methods are presented together. Scores, in line form (UNC) or dot form (WNC), are plotted against residue number. For HLA-DQ and HLA-DR results, the center line represents the mean score and the shaded area represents ±1 standard deviation. For IAd NetMHC, IAd SMM, and IEd SMM results, lines represent the mean score.
(PDF)

S3 Fig Combined MHC epitope analysis results for all immunogens/benchmarks. UNC results for all isotypes / prediction methods are plotted together for easier comparison. Scores are plotted in line form against residue number. For HLA-DQ and HLA-DR results, the shaded area represents the mean score ±1 standard deviation. For IAd NetMHC, IAd SMM, and IEd SMM results, lines represent the mean score. Shaded areas representing standard deviation could not be incorporated with the IAd and IEd results due to lack of isotype diversity (these plots summarize a single immunogen / haplotype prediction run).
(PDF)

S4 Fig Design and assessment of IAd- and IEd-specific UCAs and UCnAs. Plots display scores for UCAs, UCnAs, and a random protein of the same length, in line form (UNC) or dot form (WNC), plotted against residue number.
(PDF)

S5 Fig UCA sequences (A) HLA-DQ, (B) HLA-DR, (C) IAd NetMHC, (D) IAd SMM, and (E) IEd SMM predictions. Chimeric proteins designed to maximize immunogenicity for HLA-DR and HLA-DQ isotypes and IAd and IEd haplotypes were constructed by concatenating the twenty highest scoring epitopes from post-prediction UNC MMAs with interspacing di-glycine-lysine linkers (for hapten attachment, KGGKGGK) flanked by cathepsin S-sensitive sequences.
(PDF)

S6 Fig UCnA sequences from (A) HLA-DQ, (B) HLA-DR, (C) IAd NetMHC, (D) IAd SMM, and (E) IEd SMM for UNC analyses. Chimeric proteins designed to minimize immunogenicity for HLA-DR and HLA-DQ isotypes and IAd and IEd haplotypes were constructed by concatenating the twenty lowest scoring epitopes from post-prediction UNC MMAs with interspacing di-glycine-lysine linkers (for hapten attachment, KGGKGGK) flanked by cathepsin S-sensitive sequences.
(PDF)

S7 Fig Sequence of the Matlab-generated, random protein that was used as a comparator when assessing UCA and UCnA immunogenicity. (PDF)

S1 Table HLA population frequency data. (PDF)

S2 Table Epitope ranking and excision results for HLA-DR, IAd (NetMHC), IAd (SMM), and IEd (SMM) predictions. (PDF)
