# Supplementary material for: Computational mining of MHC class II epitopes for the development of universal immunogenic proteins
Source: PLoS One. 2022 Mar 29;17(3):e0265644. doi: 10.1371/journal.pone.0265644 (PMC8963548; doi:10.1371/journal.pone.0265644)
Supplement: S1 Fig — Epitope scoring (UNC) and anchor residue identification (WNC) results for individual isotypes / prediction methods are presented side-by-side. Scores, in line form (UNC) or dot form (WNC), are plotted against residue number. For HLA-DQ and HLA-DR results, the center line represents the mean score and the shaded area represents ±1 standard deviation. For IAd NetMHC, IAd SMM, and IEd SMM results, lines represent the mean score. (PDF) [file pone.0265644.s001.pdf]

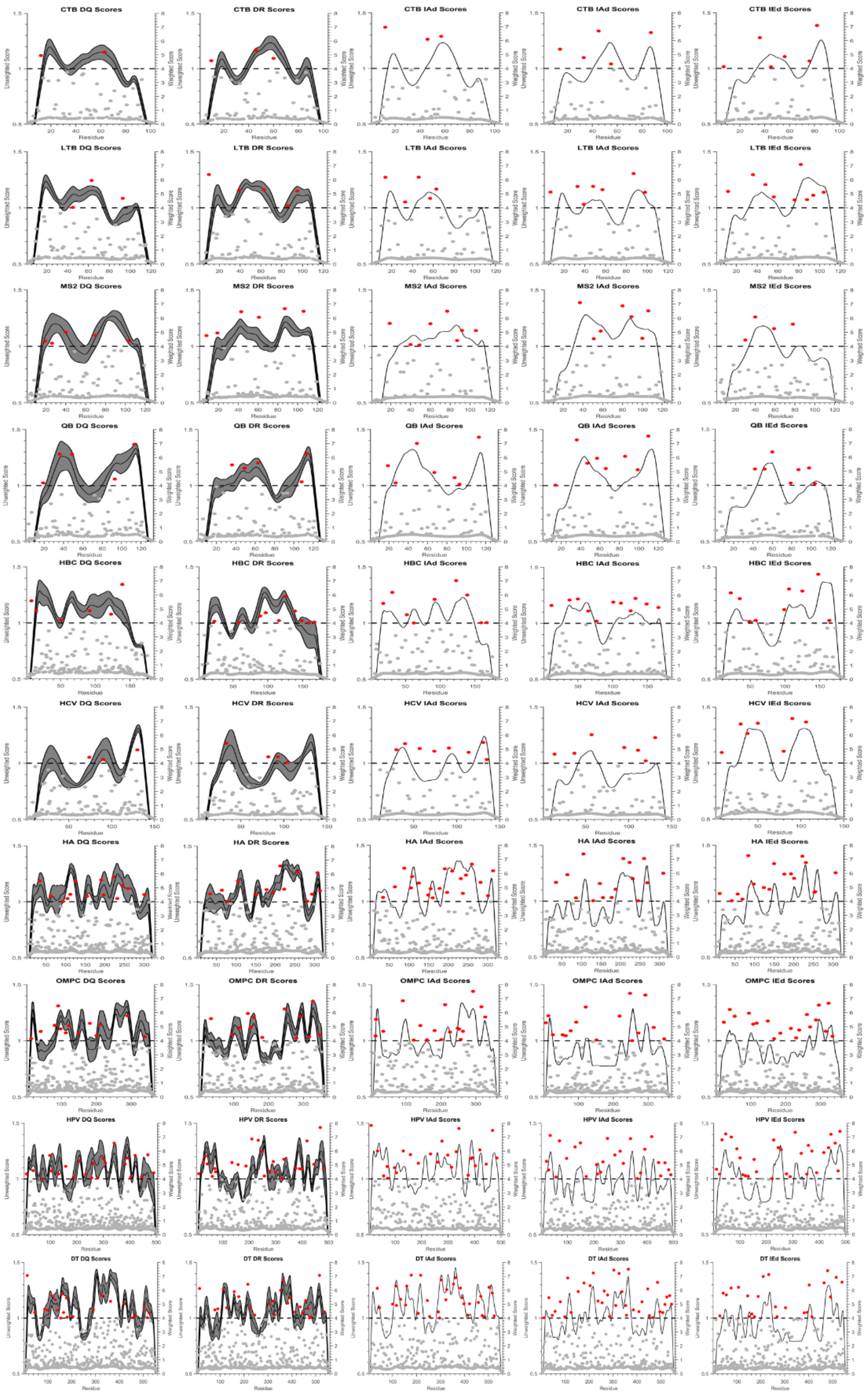
CTB DQ Scores
CTB DR Scores
CTB IAd Scores
CTB IAd Scores
CTB IEd Scores
LTB DQ Scores
LTB DR Scores
LTB IAd Scores
LTB IAd Scores
LTB IEd Scores
MS2 DQ Scores
MS2 DR Scores
MS2 IAd Scores
MS2 IAd Scores
MS2 IEd Scores
QB DQ Scores
QB DR Scores
QB IAd Scores
QB IAd Scores
QB IEd Scores
HBC DQ Scores
HBC DR Scores
HBC IAd Scores
HBC IAd Scores
HBC IEd Scores
HCV DQ Scores
HCV DR Scores
HCV IAd Scores
HCV IAd Scores
HCV IEd Scores
HA DQ Scores
HA DR Scores
HA IAd Scores
HA IAd Scores
HA IEd Scores
OMPC DQ Scores
OMPC DR Scores
OMPC IAd Scores
OMPC IAd Scores
OMPC IEd Scores
HPV DQ Scores
HPV DR Scores
HPV IAd Scores
HPV IAd Scores
HPV IEd Scores
DT DQ Scores
DT DR Scores
DT IAd Scores
DT IAd Scores
DT IEd Scores
Unweighted Score
Weighted Score
Residue

S1 Fig. MHC epitope analysis results for immunogens / benchmarks 1-10. Epitope scoring (UNC) and anchor residue identification (WNC) results for individual isotypes / prediction methods are presented side-by-side. Scores, in line form (UNC) or dot form (WNC), are plotted against residue number. For HLA-DQ and HLA-DR results, the center line represents the mean score and the shaded area represents ±1 standard deviation. For IAd NetMHC, IAd SMM, and IEd SMM results, lines represent the mean score.
