# Supplementary material for: Computational mining of MHC class II epitopes for the development of universal immunogenic proteins
Source: PLoS One. 2022 Mar 29;17(3):e0265644. doi: 10.1371/journal.pone.0265644 (PMC8963548; doi:10.1371/journal.pone.0265644)
Supplement: S2 Fig — Epitope scoring (UNC) and anchor residue identification (WNC) results for individual isotypes / prediction methods are presented together. Scores, in line form (UNC) or dot form (WNC), are plotted against residue number. For HLA-DQ and HLA-DR results, the center line represents the mean score and the shaded area represents ±1 standard deviation. For IAd NetMHC, IAd SMM, and IEd SMM results, lines represent the mean score. (PDF) [file pone.0265644.s002.pdf]

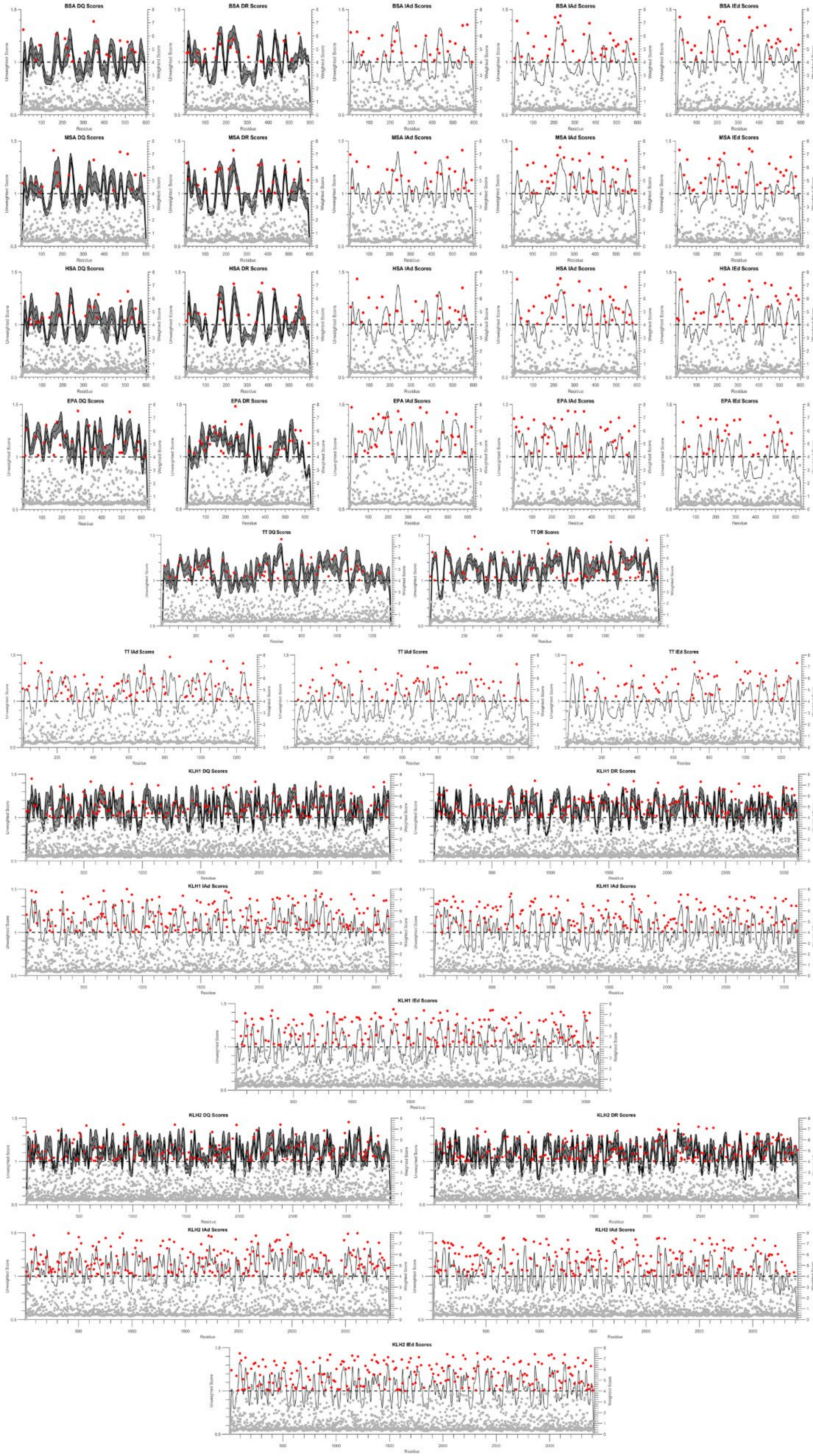
BSA DQ Scores
BSA DR Scores
BSA IAd Scores
BSA IAd Scores
BSA IEd Scores
MSA DQ Scores
MSA DR Scores
MSA IAd Scores
MSA IAd Scores
MSA IEd Scores
HSA DQ Scores
HSA DR Scores
HSA IAd Scores
HSA IAd Scores
HSA IEd Scores
EPA DQ Scores
EPA DR Scores
EPA IAd Scores
EPA IAd Scores
EPA IEd Scores
TT DQ Scores
TT DR Scores
TT IAd Scores
TT IAd Scores
TT IEd Scores
KLH1 DQ Scores
KLH1 DR Scores
KLH1 IAd Scores
KLH1 IAd Scores
KLH1 IEd Scores
KLH2 DQ Scores
KLH2 DR Scores
KLH2 IAd Scores
KLH2 IAd Scores
KLH2 IEd Scores
Unweighted Score
Weighted Score
Residue

S2 Fig. MHC epitope analysis results for immunogens / benchmarks 11-17. Epitope scoring (UNC) and anchor residue identification (WNC) results for individual isotypes / prediction methods are presented together. Scores, in line form (UNC) or dot form (WNC), are plotted against residue number. For HLA-DQ and HLA-DR results, the center line represents the mean score and the shaded area represents ±1 standard deviation. For IAd NetMHC, IAd SMM, and IEd SMM results, lines represent the mean score.
