# Supplementary material for: Computational mining of MHC class II epitopes for the development of universal immunogenic proteins
Source: PLoS One. 2022 Mar 29;17(3):e0265644. doi: 10.1371/journal.pone.0265644 (PMC8963548; doi:10.1371/journal.pone.0265644)
Supplement: S3 Fig — UNC results for all isotypes / prediction methods are plotted together for easier comparison. Scores are plotted in line form against residue number. For HLA-DQ and HLA-DR results, the shaded area represents the mean score ±1 standard deviation. For IAd NetMHC, IAd SMM, and IEd SMM results, lines represent the mean score. Shaded areas representing standard deviation could not be incorporated with the IAd and IEd results due to lack of isotype diversity (these plots summarize a single immunogen / haplotype prediction run). (PDF) [file pone.0265644.s003.pdf]

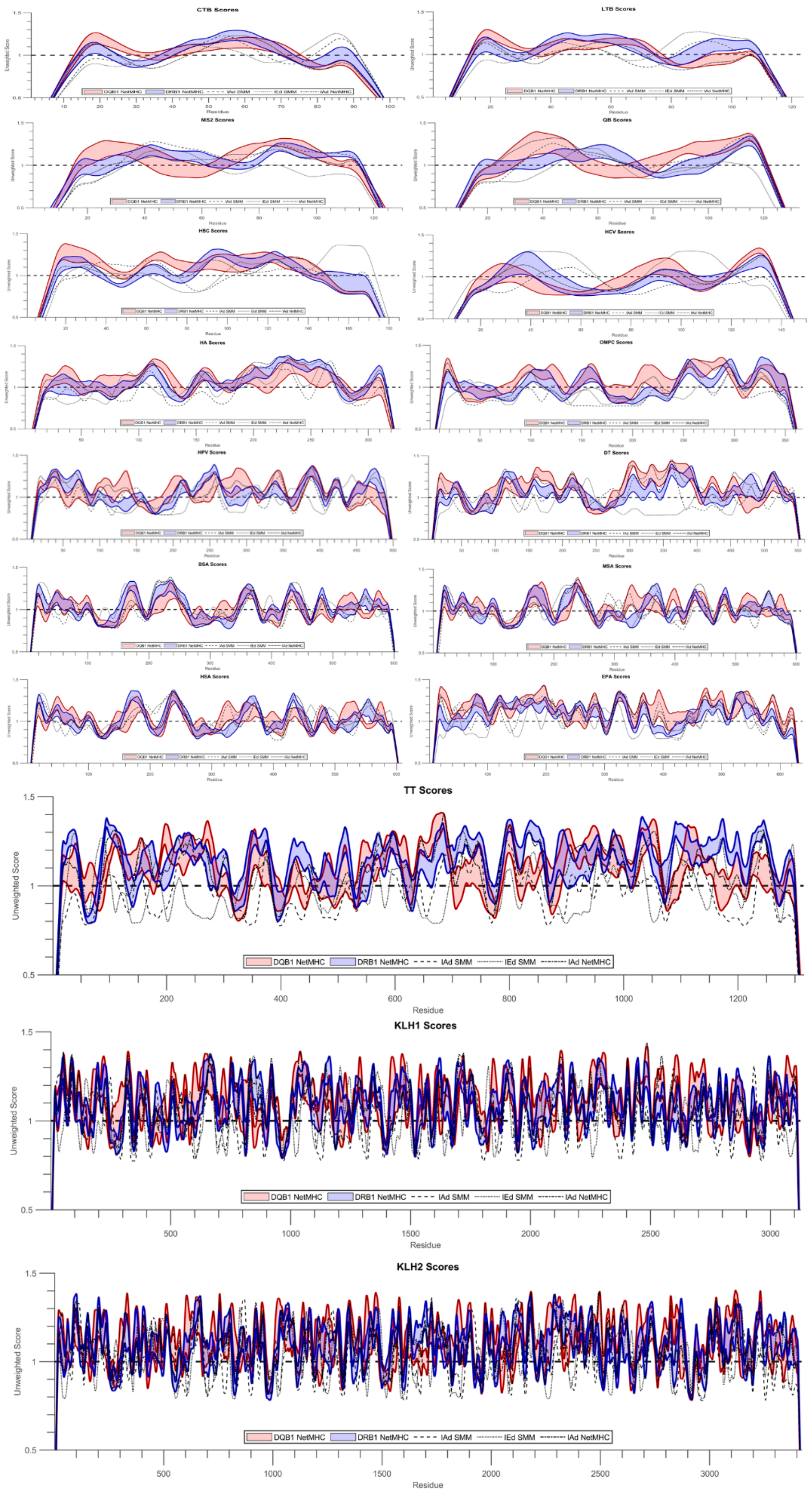

CTB Scores
LTB Scores
MS2 Scores
QB Scores
HBC Scores
HCV Scores
HA Scores
OMPC Scores
HPV Scores
DT Scores
BSA Scores
MSA Scores
HSA Scores
EPA Scores
TT Scores
KLH1 Scores
KLH2 Scores
Unweighted Score
Residue
DQB1 NetMHC
DRB1 NetMHC
IAd SMM
IEd SMM
IAd NetMHC

S3 Fig. Combined MHC epitope analysis results for all immunogens / benchmarks. UNC results for all isotypes / prediction methods are plotted together for easier comparison. Scores are plotted in line form against residue number. For HLA-DQ and HLA-DR results, the shaded area represents the mean score ±1 standard deviation. For IAd NetMHC, IAd SMM, and IEd SMM results, lines represent the mean score. Shaded areas representing standard deviation could not be incorporated with the IAd and IEd results due to lack of isotype diversity (these plots summarize a single immunogen / haplotype prediction run).
