# Supplementary material for: Computational mining of MHC class II epitopes for the development of universal immunogenic proteins
Source: PLoS One. 2022 Mar 29;17(3):e0265644. doi: 10.1371/journal.pone.0265644 (PMC8963548; doi:10.1371/journal.pone.0265644)
Supplement: S4 Fig — Plots display scores for UCAs, UCnAs, and a random protein of the same length, in line form (UNC) or dot form (WNC), plotted against residue number. (PDF) [file pone.0265644.s004.pdf]

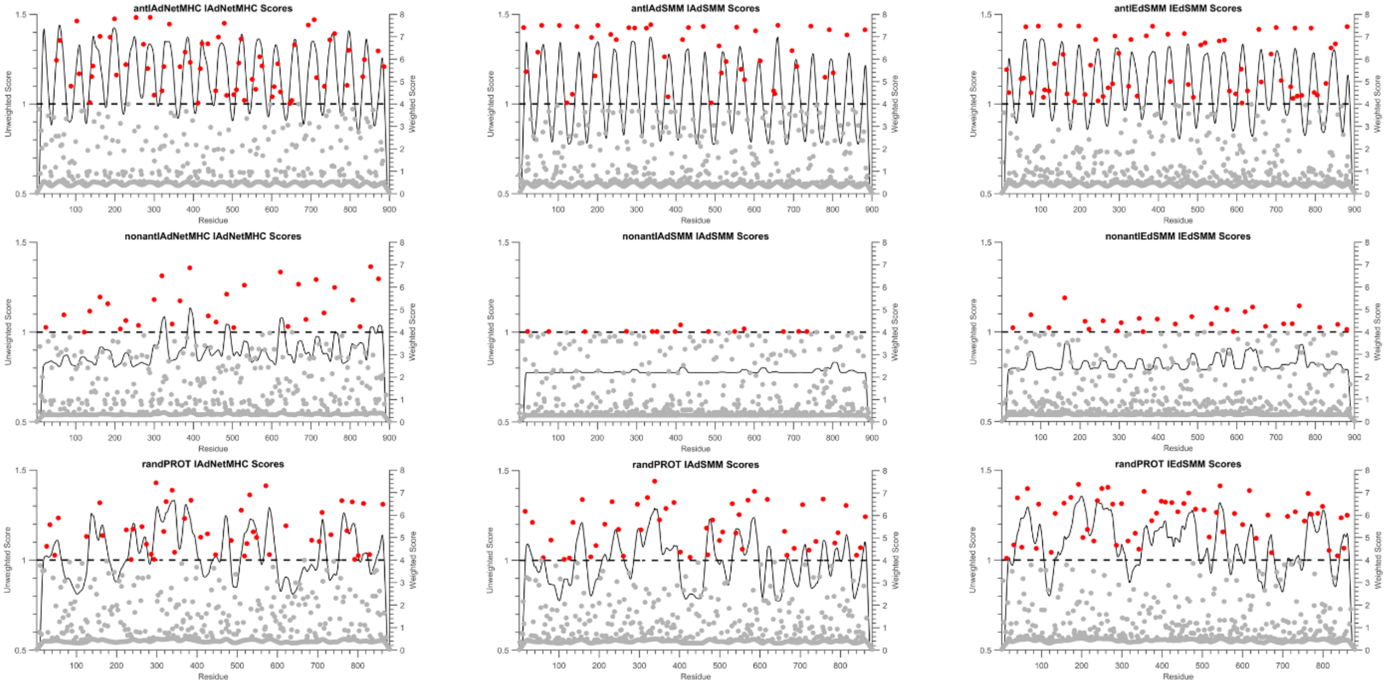

S4 Fig. Design and assessment of IAd- and IEd-specific UCAs and UCnAs. Plots display scores for UCAs, UCnAs, and a random protein of the same length, in line form (UNC) or dot form (WNC), plotted against residue number.
