# Supplementary material for: Computational mining of MHC class II epitopes for the development of universal immunogenic proteins
Source: PLoS One. 2022 Mar 29;17(3):e0265644. doi: 10.1371/journal.pone.0265644 (PMC8963548; doi:10.1371/journal.pone.0265644)
Supplement: S5 Fig — Chimeric proteins designed to maximize immunogenicity for HLA-DR and HLA-DQ isotypes and IAd and IEd haplotypes were constructed by concatenating the twenty highest scoring epitopes from post-prediction UNC MMAs with interspacing di-glycine-lysine linkers (for hapten attachment, KGGKGGK) flanked by cathepsin S-sensitive sequences. (PDF) [file pone.0265644.s005.pdf]

(A) MVAQSIALSSLMVAQAIPLVGELVDIGGVVRGGKGGKGGKGGVVRGGLWLGGTETYSMSSLAFSAYDPVFMIGGVVRGGKGGKGGKGGVVRGGLKYALSSLQAD
TSADGFAAIASFHGGGVVRGGKGGKGGKGGVVRGGVLLLEYIPEITLPVIAALSIAESSTGGVVRGGKGGKGGKGGVVRGGLRNQPRVFAGFVLSGIYTSANVKIYGG
VVRGGKGGKGGKGGVVRGGGYVFVGYHGTFLEAAQSIVFGGVRAGGVVRGGKGGKGGKGGVVRGGIPYWDWTKSMIALPAFFADSSNSNPGGVVRGGKGGKG
GKGGVVRGGESMKADHSSDGFQAIASFHALPPLCGGVVRGGKGGKGGKGGVVRGGDVPDYASLRSLVASSGTLEFITEGFGGVVRGGKGGKGGKGGVVRGGEDR
IYAGFLLAGIRTSANVDIFIKTGGVVRGGKGGKGGKGGVVRGGRAAKERTFASFILSGFGGSANVVVYGGVVRGGKGGKGGKGGVVRGGQEHSRVFAGFLLEGFGT
SATVDFQVGGVVRGGKGGKGGKGGVVRGGSQDLDAIWRGFYIAGDPALAYGYAQGGVVRGGKGGKGGKGGVVRGGKEERTFAAFLLHGFGASADVSFDVCGG
VVRGGKGGKGGKGGVVRGGGERAFKAWAVARLSQTFPNADFAEIGGVVRGGKGGKGGKGGVVRGGLARDATFFVRAHESNEMQPTLAISHGGVVRGGKGGKG
GKGGVVRGGYEIAHNYIHALVGGAQPYGMASLRYGGVVRGGKGGKGGKGGVVRGGKQEIYMQHTYPISAEELFTFGGQDAGGVVRGGKGGKGGKGGVVRGGW
ANKAQNFEAVAQYQFDFGLRPSLAGGVVRGGKGGKGGKGGVVRGGEFIKRFGDGASRVVLSLPFAEGSSS

(B) MDRFLQTMVKLFNRIKNNVAGEALLDGGVVRGGKGGKGGKGGVVRGGTQSKNILMQYIKANSKFIGITELKKGGVVRGGKGGKGGKGGVVRGGHHSNVDRLWA
VWQALQMRRHKPYRAGGVVRGGKGGKGGKGGVVRGGPNIGSRPWVRGLSSRISIYWTIVKPGGVVRGGKGGKGGKGGVVRGGFYLHHSNVDRLWAIWQALQI
RRGKSGGVVRGGKGGKGGKGGVVRGGGIPLYKKMEAVKLRDLKTYSVQLKLGGVVRGGKGGKGGKGGVVRGGKAMININIFMRESSRSFLVNQMINEGGVVRG
GKGGKGGKGGVVRGGQFEVVHNAIHYLVGGRQVYALSSQHGGVVRGGKGGKGGKGGVVRGGIHDLRNQPRVFAGFVLSGIYTSANVGGVVRGGKGGKGGKGG
VVRGGFPLWHRLYVVQLERALIRKKATISIGGVVRGGKGGKGGKGGVVRGGVFPHWHRLYTLQMDMALLSHGSAVAGGVVRGGKGGKGGKGGVVRGGLGGTKE
MAWAYNRLFKYDITHALHDGGVVRGGKGGKGGKGGVVRGGDKFNAYLANKWVFITITNDRLSSANGGVVRGGKGGKGGKGGVVRGGTTYDPIFFLHRSNTDRL
WAIWQALQGGVVRGGKGGKGGKGGVVRGGQTEMSFVFDRLYKLDITKALKKNGVGGVVRGGKGGKGGKGGVVRGGSLFFYLRREQMFVRHLFNRAGAVGEGG
VVRGGKGGKGGKGGVVRGGLFYIHHSQTDRIWAIWQSLQRFRGLGGVVRGGKGGKGGKGGVVRGGNQYVSIDKFRIFCKALNPKEIEKLYGGVVRGGKGGKGGK
GGVVRGGYLHHSNTDRIWAIWQALQKYRGFQYGGVVRGGKGGKGGKGGVVRGGPVQRLVALYLAARLSWNQVDQVIRN

(C) MESNEMQPTLAISHAGVSVVMAQAQPGGVVRGGKGGKGGKGGVVRGGEEIVAQSIALSSLMVAQAIPLVGELGGVVRGGKGGKGGKGGVVRGGMKADHSSD
GFQAIASFHALPPLCPSGGVVRGGKGGKGGKGGVVRGGQLSLVKALESMKADHSSDGFQAIASGGVVRGGKGGKGGKGGVVRGGFFIKVSVTAVNGTVLPASILH
APTIGGVVRGGKGGKGGKGGVVRGGIPNIGSRPWVRGLSSRISIYWTIVKGGVVRGGKGGKGGKGGVVRGGVWQALQMRRHKPYRAHCAISLEHMHGGVVRGG
KGGKGGKGGVVRGGALLRVYVPRSSLPGFYRTGLTLAAPGGVVRGGKGGKGGKGGVVRGGRCASIQKFGERALKAWSVARLSQKFGGVVRGGKGGKGGKGGVV
RGGRDLASLKSAMRSLQADDGVNGYQAIGGVVRGGKGGKGGKGGVVRGGSLKYALSSLQADTSADGFAAIASFHGGVVRGGKGGKGGKGGVVRGGPHWHRLY
TLQMDMALLSHGSAVAIPGGVVRGGKGGKGGKGGVVRGGEIAHNYIHALVGGAQPYGMASLRYTGGVVRGGKGGKGGKGGVVRGGETMREKVLASSARQRLR
CASIQKFGGGVVRGGKGGKGGKGGVVRGGVTGTNPVFAGANYAAWAVNVAQVIDGGVVRGGKGGKGGKGGVVRGGLKDAMRAVMADHGPNGYQAIAAFH
GGGVVRGGKGGKGGKGGVVRGGKVEVHGVNKTALPSSAIPAPTIIYSGGVVRGGKGGKGGKGGVVRGGLLEYIPEITLPVIAALSIAESSTQKGGVVRGGKGGKGGK
GGVVRGGDNLEKTTAALSILPGIGSVMGIADGGGVVRGGKGGKGGKGGVVRGGSFPLWHRLYVVQLERALIRKKATIS

(D) MRLWAVWQALQMRRHKPYRAHCAISLGGVVRGGKGGKGGKGGVVRGGLRCASIQKFGERALKAWSVARLSQKGGVVRGGKGGKGGKGGVVRGGLDKRQQLS
LVKALESMKADHSSDGFGGVVRGGKGGKGGKGGVVRGGCASLQKFGERAFKAWAVARLSQRFPGGVVRGGKGGKGGKGGVVRGGRMELSELTERDLASLKSA
MRSLQADGGVVRGGKGGKGGKGGVVRGGQLERALIRKKATISIPYWDWTSELTGGVVRGGKGGKGGKGGVVRGGRKIRKAVDSLTVEEQTSLRRAMADLGGVV
RGGKGGKGGKGGVVRGGVDRLWAIWQALQIRRGKSYKAHCASGGVVRGGKGGKGGKGGVVRGGESNEMQPTLAISHAGVSVVMAQAQPGGVVRGGKGGKG
GKGGVVRGGLRKALKNMQADDSPDGYQAIASFHAGGVVRGGKGGKGGKGGVVRGGSERDIGSLKYALSSLQADTSADGFAGGVVRGGKGGKGGKGGVVRGGY
TIEMGDELLAKLARDATFFVRAHEGGVVRGGKGGKGGKGGVVRGGVQRLVALYLAARLSWNQVDQVIRNAGGVVRGGKGGKGGKGGVVRGGSQTDRIWAIWQ
ALQEHRGLSGKEAHGGVVRGGKGGKGGKGGVVRGGTEEIVAQSIALSSLMVAQAIPLVGEGGVVRGGKGGKGGKGGVVRGGSVEHLTQEETLDLQAALRELQMD
SSGGVVRGGKGGKGGKGGVVRGGWHRLFVKQMEDALAAHGAHIGIPYWGGVVRGGKGGKGGKGGVVRGGGSHQADEYREAVTSASHIRKNIRDLGGVVRGG
KGGKGGKGGVVRGGWHRLYTKQMEDALTAHGARVGLPYWGGVVRGGKGGKGGKGGVVRGGWHRLYTLQMDMALLSHGSAVAIPYW

(E) MSARQRLRCASIQKFGERALKAWSVAGGVVRGGKGGKGGKGGVVRGGASVIREHARVKFDKVPRSRLIRKNVGGVVRGGKGGKGGKGGVVRGGVVRRRDRGRS
PRRRTPSPRRRRSPSGGVVRGGKGGKGGKGGVVRGGFVDKIWAVWQALQKKRKRPYHKADCGGVVRGGKGGKGGKGGVVRGGFPLWHRLYVVQLERALIRKKA
TISIGGVVRGGKGGKGGKGGVVRGGNVDRLWAVWQALQMRRHKPYRAHCAGGVVRGGKGGKGGKGGVVRGGKCASLQKFGERAFKAWAVARLSQRFGGVV
RGGKGGKGGKGGVVRGGYLLPRRGPRLGVRATRKTSERSQPRGGVVRGGKGGKGGKGGVVRGGLYEIARRHPYFYAPELLFFAKRYKAGGVVRGGKGGKGGKGG
VVRGGTFPHWHRAYVVHMERALQTKRRTSGGGVVRGGKGGKGGKGGVVRGGHGMSIFPHWHRLHTIQFERALKKHGGGVVRGGKGGKGGKGGVVRGGFFLH
RSNTDRLWAIWQALQKYRGKPGGVVRGGKGGKGGKGGVVRGGGWLLSPRGSRPSWGPTDPRRRSRNLGGVVRGGKGGKGGKGGVVRGGHATTDRIWAIWQ
DLQRFRKRPYREAGGVVRGGKGGKGGKGGVVRGGSGLDRLWIIWQELQKLRKKPYNAAKGGVVRGGKGGKGGKGGVVRGGSKTHISVNGRKIRMRCRAIDGDV
TFGGVVRGGKGGKGGKGGVVRGGFLEKRYEKWIEVYKLVKAKWLGTVNGGVVRGGKGGKGGKGGVVRGGPWQFDRLYKYDITKTLKDMKLRYDDGGVVRGGK
GGKGGKGGVVRGGHSNVDRLWAIWQALQIRRGKSYKAHGGVVRGGKGGKGGKGGVVRGGGSSVAVPYWDWTKRIEHLPHLISDA

S5 Fig. UCA sequences (A) HLA-DQ, (B) HLA-DR, (C) IAd NetMHC, (D) IAd SMM, and (E) IEd SMM predictions. Chimeric proteins designed to maximize immunogenicity for HLA-DR and HLA-DQ isotypes and IAd and IEd haplotypes were constructed by concatenating the twenty highest scoring epitopes from post-prediction UNC MMAs with interspacing di-glycine-lysine linkers (for hapten attachment, KGGKGGK) flanked by cathepsin S-sensitive sequences.
