# Supplementary material for: Computational mining of MHC class II epitopes for the development of universal immunogenic proteins
Source: PLoS One. 2022 Mar 29;17(3):e0265644. doi: 10.1371/journal.pone.0265644 (PMC8963548; doi:10.1371/journal.pone.0265644)
Supplement: S6 Fig — Chimeric proteins designed to minimize immunogenicity for HLA-DR and HLA-DQ isotypes and IAd and IEd haplotypes were constructed by concatenating the twenty lowest scoring epitopes from post-prediction UNC MMAs with interspacing di-glycine-lysine linkers (for hapten attachment, KGGKGGK) flanked by cathepsin S-sensitive sequences. (PDF) [file pone.0265644.s006.pdf]

(A) MWRYDRVYKYEITQQLHDLDLHVGDNGGVVRGGKGGKGGKGGVVRGGCCHGDLLECADDRADLAKYICDNQDGGVVRGGKGGKGGKGGVVRGGDCAQELLH
QKMEPFSWEDNDIPLTNGGVVRGGKGGKGGKGGVVRGGKVFDEFKPLVEEPQNLIKQNCELFEGGVVRGGKGGKGGKGGVVRGGECCHGDLLECADDRADLAK
YICENQGGVVRGGKGGKGGKGGVVRGGYLLPRRGPRLGVRATRKTSERSQPRGGVVRGGKGGKGGKGGVVRGGCKPPIGEHWGKGSPCTNVAVNPGDCGGVV
RGGKGGKGGKGGVVRGGLTFIAEKNSFSEEPFQDEIVSYNTKGGVVRGGKGGKGGKGGVVRGGTLIDALLGDPHCDVFQDETWDLFVEGGVVRGGKGGKGGKGG
VVRGGIDSYKQIYQQKYQFDKDSNGQYIVNGGVVRGGKGGKGGKGGVVRGGEKDAIPENLPPLTADFAEDKDVCKNGGVVRGGKGGKGGKGGVVRGGCEKQEP
ERNECFLSHKDDSPDLPKLGGVVRGGKGGKGGKGGVVRGGGKLNIYYRRLYNGLKFIIKRYTPNNGGVVRGGKGGKGGKGGVVRGGNYKVQVKIQNPTACTANGS
CDPSVTGGVVRGGKGGKGGKGGVVRGGQFDRLYKYDITKTLKDMKLRYDDTFGGVVRGGKGGKGGKGGVVRGGCTKQEPERNECFLQHKDDNPSLPPFGGVVR
GGKGGKGGKGGVVRGGIGGAISSSKRTDAQNTAAYIGNGDRGGVVRGGKGGKGGKGGVVRGGTKVNKECCHGDLLECADDRAELAKYGGVVRGGKGGKGGKG
GVVRGGFKYDITEVANRLNMHHDDTFNFRLEGGVVRGGKGGKGGKGGVVRGGQKGIQKPKSGTQGNYDDDWKGFYST

(B) MLGVRATRKTSERSQPRGRRQPIPKAGGVVRGGKGGKGGKGGVVRGGDLLECADDRADLAKYICDNQDTISSGGVVRGGKGGKGGKGGVVRGGDAIPENLPPLT
ADFAEDKDVCKNYQGGVVRGGKGGKGGKGGVVRGGDTMPADLPAIAADFVEDQEVCKNYAGGVVRGGKGGKGGKGGVVRGGCCHGDLLECADDRADLAKYIC
ENQDGGVVRGGKGGKGGKGGVVRGGTPADLFDYCELHYDYDTLNLNGMTPGGVVRGGKGGKGGKGGVVRGGVENDEMPADLPSLAADFVESKDVCKGGVVR
GGKGGKGGKGGVVRGGPGYVDSIQKGIQKPKSGTQGNYDDDGGVVRGGKGGKGGKGGVVRGGLHYFSDNKDVDGDQTYMRLGFKGETGGVVRGGKGGKGGK
GGVVRGGDALRQNGDGVGGSITYDYEGFGIGGGGVVRGGKGGKGGKGGVVRGGEPERNECFLQHKDDNPSLPPFERPEGGVVRGGKGGKGGKGGVVRGGNPP
SSLIEGASEYYDPNYLRTDSDKGGVVRGGKGGKGGKGGVVRGGLPKLDELRDEGKASSAKQRLKCASLGGVVRGGKGGKGGKGGVVRGGICRPDQSCQEAGYFSVL
GGSSEMPWGGVVRGGKGGKGGKGGVVRGGKQEPERNECFLQHKDDNPNLPRLVRGGVVRGGKGGKGGKGGVVRGGYVDPEDGVEKHNPWFDGHIDTVDKT
GGVVRGGKGGKGGKGGVVRGGLVEVSRNLGKVGSKCCKHPEAKRMPGGVVRGGKGGKGGKGGVVRGGKVNKECCHGDLLECADDRAELAKYMGGVVRGGKG
GKGGKGGVVRGGVTDQLTGYGQWEYQIQGNSAENENNGGVVRGGKGGKGGKGGVVRGGGAPYNLNDHTHDFSKPEDTFDYQKF

(C) MDCCEKQEPERNECFLSHKDDSPDLPGGVVRGGKGGKGGKGGVVRGGADCCTKQEPERNECFLQHKDDNPSLGGVVRGGKGGKGGKGGVVRGGKECCHGDLL
ECADDRADLAKYICDNGGVVRGGKGGKGGKGGVVRGGQKPKSGTQGNYDDDWKGFYSTDNKYGGVVRGGKGGKGGKGGVVRGGPTIEHHGGDHHGGDTSG
HDHSERHDGGVVRGGKGGKGGKGGVVRGGMADCCAKQEPERNECFLQHKDDNPNGGVVRGGKGGKGGKGGVVRGGHTECCHGDLLECADDRADLAKYICEG
GVVRGGKGGKGGKGGVVRGGIPLTNEHSTPADLFDYCELHYDYDTGGVVRGGKGGKGGKGGVVRGGEVPGSQHIDSQKKAIERMKDTLRITGGVVRGGKGGKGG
KGGVVRGGYEFGTKPEDFNPPSSLIEGASEYYDGGVVRGGKGGKGGKGGVVRGGRLGFKGETQVTDQLTGYGQWEYQIQGGVVRGGKGGKGGKGGVVRGGVFD
EFKPLVEEPQNLIKQNCELFEQGGVVRGGKGGKGGKGGVVRGGDYEYKIYSGPDKEQIADEINNLKNKGGVVRGGKGGKGGKGGVVRGGNLNDHTHDFSKPEDTF
DYQKFGYIYGGVVRGGKGGKGGKGGVVRGGNLNKRTQEFSKPEDTFDYHRFGYEYGGVVRGGKGGKGGKGGVVRGGSKIEFEGENVHTKRDINRDRLFQGSGGV
VRGGKGGKGGKGGVVRGGGVDTTRSPRDKLFNDPERGSESFFYGGVVRGGKGGKGGKGGVVRGGFDKSDNNDEATKTHATPHDGFEYQNGGVVRGGKGGKGG
KGGVVRGGLTKVNKECCHGDLLECADDRAELAKGGVVRGGKGGKGGKGGVVRGGLPPLTADFAEDKDVCKNYQEAKDAF

(D) MTNNGRDALRQNGDGVGGSITYDYEGGGVVRGGKGGKGGKGGVVRGGFVYVCIPDDNDRNDDHCEKAGDFFVGGVVRGGKGGKGGKGGVVRGGYVSYNNN
EHIVGYPKDGNAFNNLDRGGVVRGGKGGKGGKGGVVRGGQGGEQNCKTKAGSFTILGGETEMPFGGVVRGGKGGKGGKGGVVRGGDSNGQYIVNEDKFQILY
NSIMYGFTGGVVRGGKGGKGGKGGVVRGGRYEFGTKPEDFNPPSSLIEGASEYYGGVVRGGKGGKGGKGGVVRGGVAVNPGDCPPLELINTVIQDGDMVDGGV
VRGGKGGKGGKGGVVRGGSVPFNVFDYKTNFNYEYDTLEFNGLGGVVRGGKGGKGGKGGVVRGGDPETGRDIPNPFIGSKIEFEGENVHGGVVRGGKGGKGGK
GGVVRGGDALLERNYPTGAEFLGDGGDISFSTGGVVRGGKGGKGGKGGVVRGGHEKYVDPEDGVEKHNPWFDGHIDTVGGVVRGGKGGKGGKGGVVRGGGLK
FIIKRYTPNNEIDSFVKSGDFIGGVVRGGKGGKGGKGGVVRGGTPIPFSYSKNLDCWVDNEEDIDVILGGVVRGGKGGKGGKGGVVRGGLLDDTIYNDTEGFNIESKD
LKSEYKGGVVRGGKGGKGGKGGVVRGGHHTDLFNQILYAFEQEDYCDFEVQFGGVVRGGKGGKGGKGGVVRGGPTIEHHGGDHHGGDTSGHDHSERHDGGVV
RGGKGGKGGKGGVVRGGKFHGSPGLCQLNGNPISCCVHGMPTGGVVRGGKGGKGGKGGVVRGGASLGLVGTHNGQIGNDPNRDILIASGGVVRGGKGGKGGK
GGVVRGGLEQENYCDFEIQFEILHNGIHSWVGGGVVRGGKGGKGGKGGVVRGGECITPNGSIPNDKPFQNVNKITYGA

(E) METADNLEKTTAALSILPGIGSVMGIGGVVRGGKGGKGGKGGVVRGGGNFIGALETTGVVLLLEYIPEITLPGGVVRGGKGGKGGKGGVVRGGDQGPNGYESIAGY
HGYPFLCPEHGEGGVVRGGKGGKGGKGGVVRGGQDETGTSVLLDQTLLALEQTDFCDFGGVVRGGKGGKGGKGGVVRGGSVSFNLLEQDPSGATTKSAMLTNLI
GGVVRGGKGGKGGKGGVVRGGVYNNWFCNQALYALEQENYCDFEIQGGVVRGGKGGKGGKGGVVRGGNLDINNDIISDISGFNSSVITYPDAGGVVRGGKGGK
GGKGGVVRGGSDEVLALEKALDDLQQDDSNQGYQAGGVVRGGKGGKGGKGGVVRGGPELATSETYLDPVTGETKNNPFHHAGGVVRGGKGGKGGKGGVVRG
GTALEHPELSELKTVTGTNPVFAGANGGVVRGGKGGKGGKGGVVRGGQALEEDNYCDFEVQYEILHNEVHALGGVVRGGKGGKGGKGGVVRGGVAVDDGFSITV
EITDVDGSPPSADLGGVVRGGKGGKGGKGGVVRGGRNALASPGSGGDLGEAIREQPEQARGGVVRGGKGGKGGKGGVVRGGFQGESGHDIKITAENTPLPIAGVL
LGGVVRGGKGGKGGKGGVVRGGALKLAIDNALSITSDGLTIRLEGGVGGVVRGGKGGKGGKGGVVRGGATWVGNNLEDPASRDLVVNYVNTNVGGVVRGGKG
GKGGKGGVVRGGFVYVCIPDDNDRNDDHCEKAGDFFVGGVVRGGKGGKGGKGGVVRGGYPDAQLVPGINGKAIHLVNNESSEVGGVVRGGKGGKGGKGGVVR
GGFNQILYAFEQEDYCDFEVQFEITHNGGVVRGGKGGKGGKGGVVRGGFIEQALLALEQTNYCDFEVQFEIMH

S6 Fig. UCnA sequences from (A) HLA-DQ, (B) HLA-DR, (C) IAd NetMHC, (D) IAd SMM, and (E) IEd SMM for UNC analyses. Chimeric proteins designed to minimize immunogenicity for HLA-DR and HLA-DQ isotypes and IAd and IEd haplotypes were constructed by concatenating the twenty lowest scoring epitopes from post-prediction UNC MMAs with interspacing di-glycine-lysine linkers (for hapten attachment, KGGKGGK) flanked by cathepsin S-sensitive sequences.
