# Supplementary material for: Computational mining of MHC class II epitopes for the development of universal immunogenic proteins
Source: PLoS One. 2022 Mar 29;17(3):e0265644. doi: 10.1371/journal.pone.0265644 (PMC8963548; doi:10.1371/journal.pone.0265644)
Supplement: S7 Fig — (PDF) [file pone.0265644.s007.pdf]

PVDHPYCFCHERGYIWEAMLEECAAVCMIDGNHGRSSKVWCMLYNVGKFVTCHSFTQAKTCRSTDSYWNSHIKKCTFMFHFQIEFIIVFDDYHPKTYEKKPEGKGRWPEL
GCEMEATPHQDLCNLFWAEQNGMYFFYLNSIPAEMGRQCCAHADTYIYAMERVMFVPQFVCNSGIWTGHWKPAFRLPECYSTPWWKKSPRHFRQPWHTHRHWCLS
YNVSSDHGYVGYKILEYRMESRKFTITFIRLKMGAHKWDNSKINMERHGWMRVFPRHIWEPYNIHLLDDRINMDCSRQSYSLIQTVFINRSHNAHNPLQGTHSLCRAMQ
MAGTCTITHQHHRSMMTAPYLANTIIDEQVASIRDDKKKGLIPHFAIIPAYIDVVGIKENWRIAWIANDLKCLQFHDLMACREFPFTYGSHRLCVGDEDSQFYHFKAHENY
WQFGNTYFPHLRQAGGCVWLFGNHKPGACTRQFRLLTIKSKPSRETSREIHHVDPKKCNEMSQSDSICWPLDDLIKTGMANWYKPNNANHLIPPTYTWICINHALRKGQ
AYTRRRNLVYVQRYIQYRGCDDCELYMKEMVPQMNTHMFFARKCVTSDLAMVYPFFCYCQNDWHERGQQNYYWWYELQMDKARHGDCEHNCMDCENCRHHNFT
GFTQEFDIGTHEVVDGPMKYLADKSWPWIGDQLCSYTYQFMSNSPTDHDWGTNTQGYEYNMALFHMAWGSPNRGQRFQQDPQPNKYHHLQPKICHCCNLAHCLEG
SWQYWAQWQTVRIFQGHAKPFHRSNQMMFKKMTYHGEFAVHMRWPMPRYNNVKGRTMDDRKYIAMSQYDSRGGGTSVFVQGCQKGYYFSPCESWMPGSRMP
YVYSTCEMQHTLRPHLIFYTRVCSMA

S7 Fig. Sequence of the Matlab-generated, random protein that was used as a comparator when assessing UCA and UCnA immunogenicity.
