# Supplementary material for: Computational mining of MHC class II epitopes for the development of universal immunogenic proteins
Source: PLoS One. 2022 Mar 29;17(3):e0265644. doi: 10.1371/journal.pone.0265644 (PMC8963548; doi:10.1371/journal.pone.0265644)
Supplement: S1 Table — (PDF) [file pone.0265644.s008.pdf]

S1 Table. HLA Population Frequency Data.

	African American			Asian / Pacific Islander			Caucasian			Hispanic / Latino			Native American			Total		
**Allele**	**Freq.**	**Rank**	**Cum. Freq.**	**Freq.**	**Rank**	**Cum. Freq.**	**Freq.**	**Rank**	**Cum. Freq.**	**Freq.**	**Rank**	**Cum. Freq.**	**Freq.**	**Rank**	**Cum. Freq.**	**Freq.**	**Rank**	**Cum. Freq.**
**DQB1*02:01**	0.21445675	1	0.21445675	0.13066803	3	0.13066803	0.21184963	1	0.21184963	0.17498520	3	0.17498520	0.16979764	2	0.16979764	0.20666206	1	0.20666206
**DQB1*03:01**	0.18508610	3	0.39954286	0.18486434	1	0.31553237	0.19619945	2	0.40804908	0.20146520	1	0.37645040	0.23701749	1	0.40681512	0.19586026	2	0.40252232
**DQB1*06:02**	0.19720615	2	0.59674900	0.03550787	10	0.35104024	0.12785276	3	0.53590184	0.07545464	6	0.45190504	0.10742286	4	0.51423798	0.12397091	3	0.52649323
**DQB1*05:01**	0.14866494	4	0.74541394	0.08092854	5	0.43196878	0.11616724	4	0.65206909	0.10773516	4	0.55964020	0.09528701	5	0.60952499	0.11547339	4	0.64196662
**DQB1*03:02**	0.03775619	7	0.78317013	0.07493012	6	0.50689890	0.10349175	5	0.75556084	0.18909041	2	0.74873061	0.14318824	3	0.75271323	0.10487334	5	0.74683996
**DQB1*06:03**	0.02628023	8	0.80945035	0.03205222	11	0.53895113	0.06467840	6	0.82023924	0.04666294	7	0.79539355	0.04362699	8	0.79634022	0.06094144	6	0.80778141
**DQB1*03:03**	0.01318804	12	0.82263839	0.10363787	4	0.64258900	0.04313609	7	0.86337534	0.01891761	10	0.81431116	0.04798582	7	0.84432604	0.04300742	7	0.85078883
**DQB1*06:04**	0.01824812	10	0.84088651	0.01851444	12	0.66110344	0.03752754	8	0.90090287	0.02794879	8	0.84225996	0.02780798	10	0.87213401	0.03552748	8	0.88631631
**DQB1*04:02**	0.07035422	5	0.91124073	0.01568920	14	0.67679264	0.02816427	10	0.92906715	0.10195355	5	0.94421350	0.06611828	6	0.93825229	0.03347996	9	0.91979626
**DQB1*05:03**	0.01720774	11	0.92844846	0.05953326	7	0.73632589	0.02851587	9	0.95758301	0.02001793	9	0.96423143	0.03018825	9	0.96844054	0.02880917	10	0.94860544
**DQB1*05:02**	0.02562848	9	0.95407694	0.05880128	8	0.79512718	0.02017834	11	0.97776135	0.01276413	11	0.97699557	0.01064016	11	0.97908070	0.02137791	11	0.96998335
**DQB1*06:01**	0.00221138	14	0.95628832	0.14064067	2	0.93576785	0.00907484	12	0.98683619	0.01151695	12	0.98851252	0.00949895	13	0.98857966	0.01390526	12	0.98388861
**DQB1*06:09**	0.04012444	6	0.99641276	0.01749519	13	0.95326303	0.00874071	13	0.99557690	0.01008323	13	0.99859574	0.01010229	12	0.99868194	0.01030867	13	0.99419728
**DRB1*07:01**	0.10054782	2	0.10054782	0.09907500	1	0.09907500	0.12966658	1	0.12966658	0.10746544	1	0.10746544	0.10269893	1	0.10269893	0.12110722	1	0.12110722
**DRB1*15:01**	0.02726906	14	0.12781687	0.08219547	4	0.18127047	0.12932397	2	0.25899054	0.06127427	5	0.16873971	0.10244313	2	0.20514206	0.10702635	2	0.22813357
**DRB1*03:01**	0.06954377	5	0.19736064	0.05635386	6	0.23762433	0.11410009	3	0.37309064	0.07021828	2	0.23895799	0.08849214	3	0.29363420	0.09881680	3	0.32695037
**DRB1*01:01**	0.02595322	15	0.22331386	0.02643606	15	0.26406039	0.08513416	4	0.45822480	0.04489203	8	0.28385002	0.06756489	4	0.36119909	0.06898665	4	0.39593702
**DRB1*11:01**	0.08454400	3	0.30785786	0.05365153	7	0.31771192	0.06351149	6	0.52173629	0.04081965	10	0.32466967	0.04448938	9	0.40568847	0.06141732	5	0.45735434
**DRB1*04:01**	0.01921546	17	0.32707332	0.00716754	24	0.32487946	0.08040343	5	0.60213971	0.01866604	18	0.34333571	0.06510911	5	0.47079757	0.05999214	6	0.51734648
**DRB1*13:01**	0.05394688	8	0.38102020	0.03182463	13	0.35670410	0.06202056	7	0.66416027	0.04568786	7	0.38902357	0.04333852	10	0.51413610	0.05614573	7	0.57349221
**DRB1*13:02**	0.07341943	4	0.45443963	0.03604044	12	0.39274454	0.04641136	8	0.71057164	0.04110303	9	0.43012660	0.03842419	11	0.55256029	0.04703741	8	0.62052963
**DRB1*04:04**	0.00813001	22	0.46256964	0.01251130	20	0.40525584	0.03408895	10	0.74466058	0.04658773	6	0.47671433	0.04738914	7	0.59994942	0.03132798	9	0.65185761
**DRB1*11:04**	0.00636926	23	0.46893891	0.00963374	22	0.41488958	0.03639585	9	0.78105643	0.03045459	12	0.50716892	0.01685003	16	0.61679946	0.03021251	10	0.68207012
**DRB1*14:01**	0.01869779	18	0.48763670	0.02210476	16	0.43699434	0.02730850	11	0.80836492	0.01817836	19	0.52534728	0.02657817	14	0.64337762	0.02489766	11	0.70696778
**DRB1*12:01**	0.03728088	11	0.52491758	0.02037562	17	0.45736996	0.01746922	13	0.82583414	0.01147894	29	0.53682622	0.01364326	19	0.65702088	0.01872526	12	0.72569304
**DRB1*09:01**	0.02966668	13	0.55458425	0.07684143	5	0.53421139	0.00981115	18	0.83564530	0.01159590	27	0.54842212	0.02560685	15	0.68262773	0.01853953	13	0.74423257
**DRB1*01:02**	0.04014112	9	0.59472538	0.00116188	35	0.53537328	0.01509289	15	0.85073819	0.03444548	11	0.58286760	0.01054100	21	0.69316872	0.01828134	14	0.76251391
**DRB1*08:01**	0.00361721	26	0.59834259	0.00174050	32	0.53711378	0.02366273	12	0.87440091	0.01091359	30	0.59378119	0.01521861	18	0.70838733	0.01808072	15	0.78059463
**DRB1*15:02**	0.00264935	28	0.60099195	0.09739959	2	0.63451337	0.00903081	23	0.88343173	0.01152781	28	0.60530899	0.00862894	26	0.71701627	0.01752194	16	0.79811657
**DRB1*04:07**	0.00388927	25	0.60488121	0.00159353	33	0.63610690	0.00973562	19	0.89316735	0.06494586	4	0.67025485	0.04518431	8	0.76220058	0.01554992	17	0.81366649
**DRB1*04:05**	0.01577253	19	0.62065374	0.05042172	8	0.68652862	0.00651698	25	0.89968432	0.02404857	14	0.69430342	0.00989842	22	0.77209900	0.01387832	18	0.82754482
**DRB1*10:01**	0.01951620	16	0.64016994	0.03719093	10	0.72371954	0.00933766	20	0.90902199	0.01613254	21	0.71043596	0.00879930	25	0.78089830	0.01382807	19	0.84137289
**DRB1*16:01**	0.00196397	31	0.64213391	0.00050708	40	0.72422663	0.01744541	14	0.92646739	0.00957491	31	0.72001087	0.00839271	27	0.78929101	0.01335840	20	0.85473129
**DRB1*13:03**	0.03324294	12	0.67537685	0.00078615	38	0.72501278	0.01202338	17	0.93849077	0.01330443	22	0.73331530	0.00960102	23	0.79889203	0.01291683	21	0.86764812
**DRB1*04:03**	0.00204328	30	0.67742013	0.03683899	11	0.76185176	0.00932012	21	0.94781090	0.01703610	20	0.75035140	0.01327973	20	0.81217176	0.01239276	22	0.88004088
**DRB1*15:03**	0.11838911	1	0.79580923	0.00016620	54	0.76201796	0.00046940	34	0.94828030	0.01164678	26	0.76199817	0.00482646	30	0.81699822	0.01223130	23	0.89227218
**DRB1*04:02**	0.00088619	34	0.79669542	0.00287673	29	0.76489469	0.01267128	16	0.96095158	0.01303068	23	0.77502885	0.00432209	31	0.82132031	0.01064303	24	0.90291520
**DRB1*08:02**	0.00091419	33	0.79760962	0.00909645	23	0.77399114	0.00072770	33	0.96167927	0.06911368	3	0.84414253	0.01682697	17	0.83814727	0.01018580	25	0.91310100
**DRB1*12:02**	0.00283084	27	0.80044046	0.08346129	3	0.85745243	0.00030031	38	0.96197958	0.00117988	41	0.84532241	0.00111617	39	0.83926344	0.00886889	26	0.92196990
**DRB1*08:04**	0.05456123	7	0.85500169	0.00044715	43	0.85789957	0.00254898	29	0.96452856	0.00923740	32	0.85455981	0.02689049	13	0.86615394	0.00794038	27	0.92991027
**DRB1*01:03**	0.00251665	29	0.85751834	0.00023127	52	0.85813085	0.00926537	22	0.97379393	0.00697161	33	0.86153142	0.00924782	24	0.87540176	0.00749306	28	0.93740334
**DRB1*03:02**	0.06305927	6	0.92057761	0.00011080	58	0.85824165	0.00037943	36	0.97417336	0.01169678	25	0.87322820	0.00319679	35	0.87859855	0.00729179	29	0.94469512
**DRB1*16:02**	0.01387990	20	0.93445750	0.01716644	18	0.87540808	0.00183949	31	0.97601285	0.02095100	15	0.89417920	0.02712835	12	0.90572689	0.00698941	30	0.95168453
**DRB1*11:02**	0.03840559	10	0.97286309	0.00012927	55	0.87553735	0.00283381	28	0.97884666	0.01260710	24	0.90678630	0.00485026	29	0.91057716	0.00692313	31	0.95860766
**DRB1*08:03**	0.00038002	40	0.97324311	0.03924501	9	0.91478236	0.00251969	30	0.98136635	0.00207808	38	0.90886439	0.00191415	37	0.91249131	0.00590654	32	0.96451420
**DRB1*11:03**	0.00069526	36	0.97393837	0.00024446	51	0.91502682	0.00688787	24	0.98825422	0.00376279	35	0.91262718	0.00332165	33	0.91581295	0.00526943	33	0.96978363
**DRB1*14:04**	0.00066403	38	0.97460240	0.03143012	14	0.94645694	0.00091084	32	0.98916505	0.00070010	44	0.91332729	0.00345687	32	0.91926982	0.00390390	34	0.97368753
**DRB1*14:06**	0.00011183	48	0.97471423	0.00197272	31	0.94842966	0.00019252	40	0.98935757	0.02798511	13	0.94131240	0.00330597	34	0.92257579	0.00383463	35	0.97752217
**DRB1*14:02**	0.00088139	35	0.97559562	0.00037342	48	0.94880307	0.00033976	37	0.98969733	0.02043414	16	0.96174654	0.06088981	6	0.98346560	0.00337005	36	0.98089221
**DRB1*04:08**	0.00058204	39	0.97617766	0.00087953	37	0.94968260	0.00404976	26	0.99374708	0.00138253	39	0.96312907	0.00221425	36	0.98567985	0.00308539	37	0.98397761
**DRB1*04:11**	0.00032769	43	0.97650535	0.00003957	73	0.94972217	0.00010748	43	0.99385456	0.02037305	17	0.98350212	0.00096446	41	0.98664431	0.00264121	38	0.98661881
**DRB1*13:05**	0.00036220	41	0.97686755	0.00010464	61	0.94982682	0.00293635	27	0.99679092	0.00353860	36	0.98704072	0.00100471	40	0.98764902	0.00248892	39	0.98910774
**DRB1*04:06**	0.00023994	46	0.97710749	0.01387379	19	0.96370060	0.00044142	35	0.99723233	0.00117443	42	0.98821515	0.00034677	45	0.98799579	0.00184327	40	0.99095100
