# Supplementary material for: Computational mining of MHC class II epitopes for the development of universal immunogenic proteins
Source: PLoS One. 2022 Mar 29;17(3):e0265644. doi: 10.1371/journal.pone.0265644 (PMC8963548; doi:10.1371/journal.pone.0265644)
Supplement: S2 Table — (PDF) [file pone.0265644.s009.pdf]

S2 Table. Epitope Ranking and Excision Results for HLA-DR, IAd (NetMHC), IAd (SMM), and IEd (SMM) Predictions.

HLA-DR Epitope Ranking and Excision Results

Protein	Epicenter	Residues	Peak Score	Cummulative Score	Anchor(s)	Anchor Location(s)
TT	97	DRFLQTMVKLFNRIKNNVAGEALLD	1.23931673	29.25771788	2	87;95
TT	834	TQSKNILMQYIKANSKFIGITELKK	1.222141744	29.1903913	3	829;838;844
KLH1	659	HHSNVDRLWAVWQALQMRRHKPYRA	1.197818092	29.12825143	3	655;663;669
HA	227	PNIGSRPWVRGLSSRISIYWTIVKP	1.210621385	29.09241572	1	223
KLH2	661	FYLHHSNVDRLWAIWQALQIRRGKS	1.223016625	29.00620111	1	668
TT	1245	GIPLYKKMEAVKLRDLKTYSVQLKL	1.186510887	28.89341984	2	1237;1245
TT	800	KAMININIFMRESSRSFLVNQMINE	1.217803608	28.6009159	2	795;805
KLH2	2288	QFEVVHNAIHYLVGGRQVYALSSQH	1.203015084	28.58346358	3	2277;2284;2293
KLH2	3227	IHDLRNQPRVFAGFVLSGIYTSANV	1.177785644	28.5308818	2	3228;3233
KLH2	98	FPLWHRLYVVQLERALIRKKATISI	1.1920528	28.36946577	1	95
KLH2	2178	VFPHWHRLYTLQMDMALLSHGSAVA	1.210229007	28.31381105	2	2170;2176
KLH1	2446	LGGTKEMAWAYNRLFKYDITHALHD	1.210726064	28.06563606	2	2440;2448
TT	1033	DKFNAYLANKWVFITITNDRLSSAN	1.174559512	28.01734886	2	1023;1033
KLH2	1066	TTYDPIFFLHRSNTDRLWAIWQALQ	1.168893874	27.8811394	1	1071
KLH1	1619	QTEMSFVFDRLYKLDITKALKKNGV	1.19328119	27.86061865	2	1617;1626
HPV	257	SLFFYLRREQMFVRHLFNRAGAVGE	1.175486958	27.69445425	2	247;257
KLH1	1900	LFYIHHSQTDRIWAIWQSLQRFRGL	1.171112455	27.64838307	2	1891;1909
TT	1093	NQYVSIDKFRIFCKALNPKEIEKLY	1.168460118	27.57953502	3	1083;1089;1101
KLH2	2731	YLHHSNTDRIWAIWQALQKYRGFQY	1.159519542	27.56618247	1	2741
EPA	327	PVQRLVALYLAARLSWNQVDQVIRN	1.184180915	27.4596692	2	316;337
KLH1	1946	GAPYNLNDHTHDFSKPEDTFDYQKF	0.748233492	19.57327539	0	-
OMPC	79	VTDQLTGYGQWEYQIQGNSAENENN	0.746625129	19.46010703	0	-
MSA	276	KVNKECCHGDLLECADDRAELAKYM	0.729899304	19.38433828	0	-
HSA	459	LVEVSRNLGKVGSKCCKHPEAKRMP	0.728592458	19.37562962	0	-
KLH1	973	YVDPEDGVEKHNPWFDGHIDTVDKT	0.765266065	19.27735761	0	-
HSA	129	KQEPERNECFLQHKDDNPNLPRLVR	0.75158902	19.26585451	0	-
KLH2	2025	ICRPDQSCQEAGYFSVLGGSSEMPW	0.718838408	19.26389585	0	-
HSA	215	LPKLDELRDEGKASSAKQRLKCASL	0.722119949	19.20756537	0	-
TT	72	NPPSSLIEGASEYYDPNYLRTDSDK	0.70618693	19.11316373	0	-
MSA	131	EPERNECFLQHKDDNPSLPPFERPE	0.760163504	19.0952844	0	-
OMPC	204	DALRQNGDGVGGSITYDYEGFGIGG	0.744765495	18.78368165	0	-
OMPC	53	LHYFSDNKDVDGDQTYMRLGFKGET	0.723260251	18.72866504	0	-
CRM197	62	PGYVDSIQKGIQKPKSGTQGNYDDD	0.718225232	18.60111301	0	-
HSA	329	VENDEMPADLPSLAADFVESKDVCK	0.692475165	18.3838731	0	-
KLH1	290	TPADLFDYCELHYDYDTLNLNGMTP	0.697513438	18.09725768	0	-
HSA	281	CCHGDLLECADDRADLAKYICENQD	0.691499166	17.99986701	0	-
MSA	332	DTMPADLPAIAADFVEDQEVCKNYA	0.652064588	17.72814104	0	-
BSA	331	DAIPENLPPLTADFAEDKDVCKNYQ	0.655413512	17.57601042	0	-
BSA	284	DLLECADDRADLAKYICDNQDTISS	0.637836037	16.82007469	0	-
HCV	53	LGVRATRKTSERSQPRGRRQPIPKA	0.62332696	16.45283751	0	-

IAd (NetMHC) Epitope Ranking and Excision Results

Protein	Epicenter	Residues	Peak Score	Cummulative Score	Anchor(s)	Anchor Location(s)
EPA	194	ESNEMQPTLAISHAGVSVVMAQAQP	1.409804768	34.01314649	2	186;199
DT	363	EEIVAQSIALSSLMVAQAIPLVGEL	1.414018877	33.77794908	2	354;363
KLH1	1716	MKADHSSDGFQAIASFHALPPLCPS	1.365889195	33.73092348	3	1713;1719;1725
KLH1	1706	QLSLVKALESMKADHSSDGFQAIAS	1.367952011	33.58693783	3	1695;1698;1713
KLH1	2485	FFIKVSVTAVNGTVLPASILHAPTI	1.417647854	33.48495437	3	2475;2486;2491
HA	226	IPNIGSRPWVRGLSSRISIYWTIVK	1.358056104	33.41123696	3	223;232;237
KLH1	669	VWQALQMRRHKPYRAHCAISLEHMH	1.353115008	33.27124066	3	663;669;679
EPA	534	ALLRVYVPRSSLPGFYRTGLTLAAP	1.349545911	33.15874102	3	523;537;541
BSA	234	RCASIQKFGERALKAWSVARLSQKF	1.36036163	33.14449496	1	231
KLH2	2127	RDLASLKSAMRSLQADDGVNGYQAI	1.341204875	33.03828056	4	2117;2121;2136;2139
KLH1	2125	SLKYALSSLQADTSADGFAAIASFH	1.354318685	32.87397558	3	2115;2130;2133
KLH2	2180	PHWHRLYTLQMDMALLSHGSAVAIP	1.365181713	32.87088235	3	2170;2176;2182
KLH1	1039	EIAHNYIHALVGGAQPYGMASLRYT	1.370551578	32.68116114	3	1032;1042;1050
BSA	218	ETMREKVLASSARQRLRCASIQKFG	1.332662075	32.67508356	3	207;212;221
DT	303	VTGTNPVFAGANYAAWAVNVAQVID	1.361640349	32.48626935	3	291;296;303
KLH1	2546	LKDAMRAVMADHGPNGYQAIAAFHG	1.342122753	32.42328523	2	2535;2550
KLH2	2494	KVEVHGVNKTALPSSAIPAPTIIYS	1.377469923	32.41908105	1	2492
TT	685	LLEYIPEITLPVIAALSIAESSTQK	1.357344446	32.4075778	2	681;685
DT	332	DNLEKTTAALSILPGIGSVMGIADG	1.34879055	32.38855308	2	322;326
KLH2	97	SFPLWHRLYVVQLERALIRKKATIS	1.334380256	32.32716208	2	95;104
BSA	337	LPPLTADFAEDKDVCKNYQEAKDAF	0.889187704	22.8777891	0	-
MSA	274	LTKVNKECCHGDLLECADDRAELAK	0.849823595	22.76363978	0	-
KLH1	1520	FDKSDNNDEATKTHATPHDGFEYQN	0.86364277	22.72315947	0	-
KLH1	2650	GVDTTRSPRDKLFNDPERGSESFFY	0.877124425	22.70816549	0	-
KLH1	1818	SKIEFEGENVHTKRDINRDRLFQGS	0.877098613	22.59232446	0	-
KLH2	1956	NLNKRTQEFSKPEDTFDYHRFGYEY	0.840257121	22.48992087	0	-
KLH1	1950	NLNDHTHDFSKPEDTFDYQKFGYIY	0.83961758	22.44470451	0	-
TT	769	DYEYKIYSGPDKEQIADEINNLKNK	0.841666655	22.28520861	0	-
HSA	409	VFDEFKPLVEEPQNLIKQNCELFEQ	0.864720853	22.27577681	0	-
OMPC	70	RLGFKGETQVTDQLTGYGQWEYQIQ	0.878512477	22.22582967	0	-
TT	62	YEFGTKPEDFNPPSSLIEGASEYYD	0.870111783	22.07546296	0	-
LTB	84	EVPGSQHIDSQKKAIERMKDTLRIT	0.83587423	21.96807784	0	-
KLH1	282	IPLTNEHSTPADLFDYCELHYDYDT	0.855398816	21.79912036	0	-
HSA	278	HTECCHGDLLECADDRADLAKYICE	0.828451991	21.35123461	0	-
HSA	123	MADCCAKQEPERNECFLQHKDDNPN	0.817674015	21.33776979	0	-
KLH2	2917	PTIEHHGGDHHGGDTSGHDHSERHD	0.81613226	21.17486224	0	-
CRM197	73	QKPKSGTQGNYDDDWKGFYSTDNKY	0.825560025	21.02226348	0	-
BSA	278	KECCHGDLLECADDRADLAKYICDN	0.820000146	20.9142337	0	-
MSA	124	ADCCTKQEPERNECFLQHKDDNPSL	0.809000415	20.67652449	0	-
BSA	125	DCCEKQEPERNECFLSHKDDSPDLP	0.809703984	20.51146298	0	-

IEd (SMM) Epitope Ranking and Excision Results

Protein	Epicenter	Residues	Peak Score	Cummulative Score	Anchor(s)	Anchor Location(s)
BSA	228	SARQRLRCASIQKFGERALKAWSVA	1.332061912	33.02945609	2	216;233
KLH1	845	ASVIREHARVKFDKVPRSRLIRKNV	1.353263143	32.34579772	3	835;841;847
HBC	160	VVRRRDRGRSPRRRTPSPRRRRSPS	1.360402139	32.18982758	2	148;164
KLH2	2326	FVDKIWAVWQALQKKRKRPYHKADC	1.328297575	32.11747997	2	2323;2331
KLH2	98	FPLWHRLYVVQLERALIRKKATISI	1.326421238	32.10244565	2	97;110
KLH1	662	NVDRLWAVWQALQMRRHKPYRAHCA	1.309714166	32.01090693	2	658;664
HSA	235	KCASLQKFGERAFKAWAVARLSQRF	1.302186782	31.91042495	2	228;234
HCV	44	YLLPRRGPRLGVRATRKTSERSQPR	1.30205986	31.57044545	2	38;50
HSA	175	LYEIARRHPYFYAPELLFFAKRYKA	1.29242438	31.47336957	1	178
KLH1	91	TFPHWHRAYVVHMERALQTKRRTSG	1.309083941	31.42064282	3	79;87;93
KLH1	2992	HGMSIFPHWHRLHTIQFERALKKHG	1.313433603	31.38426934	2	2984;2994
KLH2	1072	FFLHRSNTDRLWAIWQALQKYRGKP	1.274854281	31.37656082	1	1071
HCV	104	GWLLSPRGSRPSWGPTDPRRRSRNL	1.30116621	31.28739018	1	106
KLH1	1482	HATTDRIWAIWQDLQRFRKRPYREA	1.311259876	31.24866808	2	1481;1483
KLH2	3143	SGLDRLWIIWQELQKLRKKPYNAAK	1.299696022	31.23869591	2	3141;3147
DT	483	SKTHISVNGRKIRMRCRAIDGDVTF	1.286389612	31.21354495	2	479;491
TT	719	FLEKRYEKWIEVYKLVKAKWLGTVN	1.283462003	31.14980806	2	712;717
KLH2	2048	PWQFDRLYKYDITKTLKDMKLRYDD	1.287711245	31.03143146	2	2041;2049
KLH2	665	HSNVDRLWAIWQALQIRRGKSYKAH	1.27735389	30.85495671	1	663
KLH1	1362	GSSVAVPYWDWTKRIEHLPHLISDA	1.261139701	30.84725123	2	1355;1374
KLH1	1850	FIEQALLALEQTNYCDFEVQFEIMH	0.805286186	21.13511302	0	-
KLH1	606	FNQILYAFEQEDYCDFEVQFEITHN	0.809839164	21.09270234	0	-
TT	921	YPDAQLVPGINGKAIHLVNNESSEV	0.841571438	21.05658874	0	-
KLH1	346	FVYVCIPDDNDRNDDHCEKAGDFFV	0.809661059	21.02383337	0	-
HBC	81	ATWVGNNLEDPASRDLVVNYVNTNV	0.801846208	20.96414189	0	-
EPA	92	ALKLAIDNALSITSDGLTIRLEGGV	0.804823272	20.83764558	0	-
DT	447	FQGESGHDIKITAENTPLPIAGVLL	0.796239873	20.808548	0	-
EPA	350	RNALASPGSGGDLGEAIREQPEQAR	0.798368095	20.76401054	0	-
KLH2	822	VAVDDGFSITVEITDVDGSPPSADL	0.790669247	20.67848405	0	-
KLH1	3084	QALEEDNYCDFEVQYEILHNEVHAL	0.803185943	20.67596798	0	-
DT	290	TALEHPELSELKTVTGTNPVFAGAN	0.805693283	20.6354513	0	-
KLH2	557	PELATSETYLDPVTGETKNNPFHHA	0.797069557	20.619233	0	-
KLH2	45	SDEVLALEKALDDLQQDDSNQGYQA	0.794696154	20.55714007	0	-
TT	900	NLDINNDIISDISGFNSSVITYPDA	0.79837486	20.50010347	0	-
KLH2	1851	VYNNWFCNQALYALEQENYCDFEIQ	0.801927935	20.38373884	0	-
TT	146	SVSFNLLEQDPSGATTKSAMLTNLI	0.792081255	20.22939822	0	-
KLH2	2261	QDETGTSVLLDQTLLALEQTDFCDF	0.791057028	20.17475965	0	-
KLH2	2969	DQGPNGYESIAGYHGYPFLCPEHGE	0.792779863	20.11803453	0	-
TT	671	GNFIGALETTGVVLLLEYIPEITLP	0.790422713	19.95316646	0	-
DT	329	ETADNLEKTTAALSILPGIGSVMGI	0.790319881	19.91495592	0	-

IAd (SMM) Epitope Ranking and Excision Results

Protein	Epicenter	Residues	Peak Score	Cummulative Score	Anchor(s)	Anchor Location(s)
KLH1	665	RLWAVWQALQMRRHKPYRAHCAISL	1.371749286	32.97323085	2	663;669
BSA	233	LRCASIQKFGERALKAWSVARLSQK	1.338946313	32.72370101	3	221;231;241
KLH1	1701	LDKRQQLSLVKALESMKADHSSDGF	1.338741132	32.70288625	3	1692;1698;1713
HSA	236	CASLQKFGERAFKAWAVARLSQRFP	1.326023092	32.59990087	2	232;245
KLH2	2118	RMELSELTERDLASLKSAMRSLQAD	1.350780344	32.42032216	2	2114;2121
KLH2	108	QLERALIRKKATISIPYWDWTSELT	1.324465055	32.33067359	2	98;112
KLH2	866	RKIRKAVDSLTVEEQTSLRRAMADL	1.34836376	32.22074784	2	856;871
KLH2	668	VDRLWAIWQALQIRRGKSYKAHCAS	1.347320496	32.00104725	3	657;668;674
EPA	194	ESNEMQPTLAISHAGVSVVMAQAQP	1.289190046	31.83720292	2	183;199
KLH2	1715	LRKALKNMQADDSPDGYQAIASFHA	1.298134058	31.82225546	2	1704;1719
KLH1	2119	SERDIGSLKYALSSLQADTSADGFA	1.306740421	31.70407818	2	2115;2124
EPA	170	YTIEMGDELLAKLARDATFFVRAHE	1.289773198	31.55505498	3	160;166;178
EPA	328	VQRLVALYLAARLSWNQVDQVIRNA	1.308365497	31.27271755	3	317;328;337
KLH2	1912	SQTDRIWAIWQALQEHRGLSGKEAH	1.278892824	31.19900037	2	1903;1918
DT	362	TEEIVAQSIALSSLMVAQAIPLVGE	1.323594427	31.04185145	3	353;356;364
KLH1	34	SVEHLTQEETLDLQAALRELQMDSS	1.256176641	31.01310002	2	26;38
KLH2	2602	WHRLFVKQMEDALAAHGAHIGIPYW	1.272617067	30.93104394	1	2596
KLH2	1273	GSHQADEYREAVTSASHIRKNIRDL	1.279025872	30.82299857	2	1268;1278
KLH1	2596	WHRLYTKQMEDALTAHGARVGLPYW	1.268922188	30.56329479	1	2590
KLH2	2182	WHRLYTLQMDMALLSHGSAVAIPYW	1.294685021	30.54971096	1	2176
HA	292	ECITPNGSIPNDKPFQNVNKITYGA	0.786352203	21.01383677	0	-
KLH2	1864	LEQENYCDFEIQFEILHNGIHSWVG	0.801795897	20.97133481	0	-
TT	1275	ASLGLVGTHNGQIGNDPNRDILIAS	0.805922858	20.77778389	0	-
KLH1	1318	KFHGSPGLCQLNGNPISCCVHGMPT	0.790628922	20.62799786	0	-
KLH2	2917	PTIEHHGGDHHGGDTSGHDHSERHD	0.787566592	20.57258527	0	-
KLH1	601	HHTDLFNQILYAFEQEDYCDFEVQF	0.787973017	20.56181923	0	-
TT	400	LLDDTIYNDTEGFNIESKDLKSEYK	0.782916771	20.53887951	0	-
TT	869	TPIPFSYSKNLDCWVDNEEDIDVIL	0.789534822	20.47109494	0	-
TT	1184	GLKFIIKRYTPNNEIDSFVKSGDFI	0.809036349	20.43260043	0	-
KLH1	970	HEKYVDPEDGVEKHNPWFDGHIDTV	0.78096418	20.42501522	0	-
EPA	424	DALLERNYPTGAEFLGDGGDISFST	0.780642097	20.32065703	0	-
KLH1	1804	DPETGRDIPNPFIGSKIEFEGENVH	0.781784085	20.29800737	0	-
KLH1	1127	SVPFNVFDYKTNFNYEYDTLEFNGL	0.782864396	20.26835735	0	-
HPV	190	VAVNPGDCPPLELINTVIQDGDMVD	0.791701951	20.20160431	0	-
TT	61	RYEFGTKPEDFNPPSSLIEGASEYY	0.785574116	20.15318307	0	-
TT	347	DSNGQYIVNEDKFQILYNSIMYGFT	0.784136523	20.13840859	0	-
KLH2	1605	QGGEQNCKTKAGSFTILGGETEMPF	0.78347929	20.05169614	0	-
TT	1211	YVSYNNNEHIVGYPKDGNAFNNLDR	0.782712112	19.93961497	0	-
KLH1	346	FVYVCIPDDNDRNDDHCEKAGDFFV	0.774852208	19.76451267	0	-
OMPC	199	TNNGRDALRQNGDGVGGSITYDYEG	0.775561605	19.62668446	0	-
